# Clioquinol and pyrrolidine dithiocarbamate complex with copper to form proteasome inhibitors and apoptosis inducers in human breast cancer cells

**DOI:** 10.1186/bcr1322

**Published:** 2005-09-20

**Authors:** Kenyon G Daniel, Di Chen, Shirley Orlu, Qiuzhi Cindy Cui, Fred R Miller, Q Ping Dou

**Affiliations:** 1 The Prevention Program, Barbara Ann Karmanos Cancer Institute, and Department of Pathology, School of Medicine, Wayne State University, Detroit, Michigan, USA; 2 The Breast Cancer Program, Barbara Ann Karmanos Cancer Institute, and Department of Pathology, School of Medicine, Wayne State University, Detroit, Michigan, USA

## Abstract

**Introduction:**

A physiological feature of many tumor tissues and cells is the tendency to accumulate high concentrations of copper. While the precise role of copper in tumors is cryptic, copper, but not other trace metals, is required for angiogenesis. We have recently reported that organic copper-containing compounds, including 8-hydroxyquinoline-copper(II) and 5,7-dichloro-8-hydroxyquinoline-copper(II), comprise a novel class of proteasome inhibitors and tumor cell apoptosis inducers. In the current study, we investigate whether clioquinol (CQ), an analog of 8-hydroxyquinoline and an Alzheimer's disease drug, and pyrrolidine dithiocarbamate (PDTC), a known copper-binding compound and antioxidant, can interact with copper to form cancer-specific proteasome inhibitors and apoptosis inducers in human breast cancer cells. Tetrathiomolybdate (TM), a strong copper chelator currently being tested in clinical trials, is used as a comparison.

**Methods:**

Breast cell lines, normal, immortalized MCF-10A, premalignant MCF10AT1K.cl2, and malignant MCF10DCIS.com and MDA-MB-231, were treated with CQ or PDTC with or without prior interaction with copper, followed by measurement of proteasome inhibition and cell death. Inhibition of the proteasome was determined by levels of the proteasomal chymotrypsin-like activity and ubiquitinated proteins in protein extracts of the treated cells. Apoptotic cell death was measured by morphological changes, Hoechst staining, and poly(ADP-ribose) polymerase cleavage.

**Results:**

When in complex with copper, both CQ and PDTC, but not TM, can inhibit the proteasome chymotrypsin-like activity, block proliferation, and induce apoptotic cell death preferentially in breast cancer cells, less in premalignant breast cells, but are non-toxic to normal/non-transformed breast cells at the concentrations tested. In contrast, CQ, PDTC, TM or copper alone had no effects on any of the cells. Breast premalignant or cancer cells that contain copper at concentrations similar to those found in patients, when treated with just CQ or PDTC alone, but not TM, undergo proteasome inhibition and apoptosis.

**Conclusion:**

The feature of breast cancer cells and tissues to accumulate copper can be used as a targeting method for anticancer therapy through treatment with novel compounds such as CQ and PDTC that become active proteasome inhibitors and breast cancer cell killers in the presence of copper.

## Introduction

Copper is an essential trace metal for animals. The amount of copper in an organism is tightly regulated [1,2]. Angiogenesis, the growth of a tumor blood supply, is essential for tumor growth, invasion, and metastasis [3-6]. It has been shown that tumors, without a blood supply, do not grow larger than 1 to 2 mm^3 ^[7]. Molecular processes of angiogenesis that require copper as an essential cofactor include stimulation of endothelial growth by tumor cytokine production (i.e., vasoendothelial growth factor), degradation of extracellular matrix proteins by metalloproteinases, and migration of endothelial cells mediated by integrins [3-12]. Consistently high levels of copper have been found in many types of human cancers, including breast, prostate, colon, lung, and brain [13-21]. Three anti-copper drugs have been tested in clinical trials [8,9], particularly tetrathiomolybdate (TM), a copper chelator, which was originally used for patients with Wilson's disease [8,11]. TM has been found to be effective in impairing the growth of mammary tumors in HER2/neu transgenic mice [22] and lung metastatic carcinoma in C557BL6/J mice [23]. In a phase I clinical trial with patients suffering from metastatic cancers, TM therapy achieved stable disease in five of six patients who were copper-deficient [11]. However, the disease advanced in some other patients before copper levels were sufficiently lowered [8,9,11]. These reports support the idea of copper control as an anticancer strategy.

Apoptosis, an evolutionarily conserved form of cell death, is the process by which a cell will actively commit suicide under tightly controlled circumstances [24]. Apoptosis occurs in two physiological stages, commitment and execution [25,26]. Activation of effector caspases leads to apoptotic execution probably through the proteolytic cleavage of important cellular proteins [27], such as poly(ADP-ribose) polymerase (PARP) [28], and the retinoblastoma protein [29-31]. Other hallmarks of apoptosis include cellular shrinkage, membrane blebbing, and DNA fragmentation [25-27].

The ubiquitin/proteasome system plays an important role in the degradation of cellular proteins. This proteolytic system involves two distinct steps, ubiquitination and degradation [32,33]. The eukaryotic proteasome contains at least three known activities, which are associated with its β subunits. These are the chymotrypsin-like (cleavage after hydrophobic residues, β5 subunit), trypsin-like (cleavage after basic residues, β2 subunit), and caspase-like or peptidyl-glutamyl peptide-hydrolyzing (cleavage after acidic residues, β1 subunit) activities [34,35]. Inhibition of the proteasomal chymotrypsin-like activity has been found to be associated with induction of apoptosis in tumor cells [36-41].

**Figure 1:**
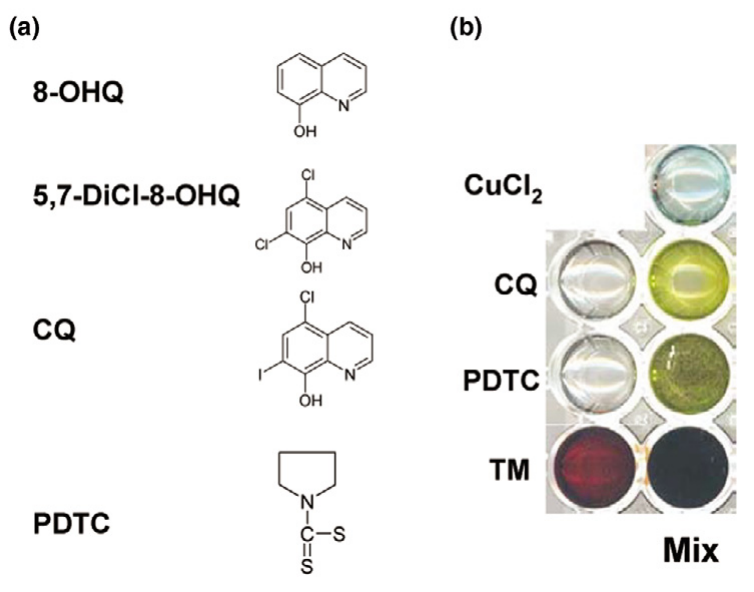
Copper complex formation as indicated by color change. **(a) **Chemical structures of compounds referenced in the text: 5,7-DiCl-8-OHQ, 5,7-dichloro-8-hydroxyquinoline; 8-OHQ, 8-hydroxyquinoline; CQ, clioquinol; PDTC, pyrrolidine dithiocarbamate. **(b) **50 mM of PDTC, CQ, or tetrathiomolybdate (TM) were mixed in a 1:1 molar ratio with CuCl_2_. All solutions were made in dimethylsulfoxide. In each case the appearance of intensified color indicated formation of a copper complex.

Most recently, we discovered that several organic-copper (but not zinc or nickel) compounds, such as bis-8-hydroxyquinoline-copper(II), potently and specifically inhibited the chymotrypsin-like activity of the proteasome *in vitro *and in human tumor cell culture [42]. Inhibition of the proteasome activity by organic copper compounds occurs very rapidly in tumor cells (15 minutes), followed by induction of apoptosis. Neither proteasome inhibition nor apoptosis were found in human normal or non-transformed cells under the same treatment. Most importantly, proteasome inhibition and apoptosis were also detected in copper-containing tumor cells treated with 8-hydroxyquinoline (8-OHQ; Fig. 1a). None of these events occurred in cells treated with either inorganic copper, ligand-treated cells that did not contain copper, or pretreatment with the closely related nickel followed by addition of the ligand [42]. We also found that 5,7-dichloro-8-hydroxyquinoline (5,7-DiCl-8-OHQ; Fig. 1a) synthesized to contain copper was a potent proteasome inhibitor and apoptosis inducer [42].

Clioquinol (5-chloro-7-iodo-8-hydroxyquinoline; CQ; Fig. 1a) belongs to the quinoline class of compounds and is structurally similar to 5,7-DiCl-8-OHQ. This class of compounds possesses an established toxicology profile with the US Pharmacopoeia [43]. During the 1950s to the 1970s, CQ was used as an antibiotic [44,45]; however, it was withdrawn due to association with subacute myelo-optic neuropathy possibly due to overdose and/or a reversible vitamin B_12 _deficiency [44,46-48]. Recently, interest in CQ has reemerged due to studies involving its use, in combination with B_12_, for treatment of Alzheimer's disease [43,49,50]. Regardless of it being a controversial compound, CQ can still serve as a model compound from which analogs could be developed that exploit its copper binding potential but avoid its negative associations. CQ is a lipophilic compound that is capable of forming stable complexes with copper(II) ions [51]. In a phase II clinical trial, CQ, at a starting concentration of 3.3 mg/kg, the same order of magnitude of treatment used in mice, was found to be well-tolerated and suitable for further study [49]. Examination of CQ in animal studies has continued to further characterize its effects [52].

Dithiocarbamates are a class of metal chelating compounds. These compounds have previously been used in the treatment of bacterial and fungal infections, and have been considered for use in the treatment of AIDS [53,54]. Pyrrolidine dithiocarbamate (PDTC; Fig. 1a) is a synthetic antioxidant and inhibitor of NF-κB that is capable of binding copper [55,56]. PDTC and other dithiocarbamates have been found to induce apoptosis in conjunction with copper in different types of cancer cells [55,57]. Previously we found a synthetic PDTC containing copper was a potent proteasome inhibitor and apoptosis inducer [42].

Here we show that CQ and PDTC are capable of binding copper, spontaneously forming new complexes that have proteasome-inhibitory and apoptosis-inducing activities to cancer but not normal/non-transformed breast cells, and that premalignant or cancer breast cells cultured to contain elevated copper are sensitive to treatment with CQ or PDTC alone. In contrast, TM-copper or TM alone had no effects in the same experiments. We propose that targeting highly elevated copper can be tumor-specific and that formation of an active anticancer proteasome inhibitory complex between CQ or PDTC and tumor cellular copper is a novel strategy that has great potential for breast cancer therapies.

## Materials and methods

### Chemicals and reagents

CQ, PDTC, disulfiram (tetraethyl thiuram disulfide), tetramethyl thiruam disulfide, methyl propyl disulfide, allyl disulfide, isopropyl disulfide, TM, CuCl_2_, dimethylsulfoxide (DMSO), bisbenzimide Hoechst No. 33258 stain, cholera toxin, hydrocortisone, 3-[4,5-dimethylthiazol-2-yl]-2,5-diphenyl-tetrazolium bromide (MTT), epidermal growth factor, and insulin were purchased from Sigma-Aldrich (St. Louis, MO, USA). F12 medium, DMEM, horse serum, penicillin, and streptomycin were purchased from Invitrogen (Carlsbad, CA, USA). Fluorogenic peptide substrate Suc-Leu-Leu-Val-Tyr-AMC (for the proteasomal chymotrypsin-like activity) was obtained from Calbiochem (San Diego, CA, USA). Mouse monoclonal antibody to human PARP was from Roche Applied Science (Indianapolis, IN, USA). Mouse monoclonal antibody to human ubiquitin was from Santa Cruz Biotechnology Inc (Santa Cruz, CA, USA).

### Cell culture and lysates preparation

MCF10A (normal-MCF10), MCF10AT1K.cl2 (premalignant-MCF10), and MCF10dcis.com (malignant-MCF10) cells were cultured as described previously [58]. Briefly, normal-MCF10 and premalignant-MCF10 cells were cultured in 1:1 F12/DMEM prepared as follows: 500 ml of media was supplemented with 5.26% (v/v) horse serum, 100 units/ml of penicillin, 100 μg/ml of streptomycin, 52.55 μg of cholera endotoxin, 5 mg insulin, 10 ml of 1 M NaHCO_3_, 10 μg of epidermal growth factor, and 250 μg hydrocortisone. Malignant-MCF10 cells were cultured in 1:1 F12/DMEM media supplemented with 5.26% (v/v) horse serum, 10 ml of 1 M NaHCO_3_, 100 units/ml of penicillin, and 100 μg/ml of streptomycin. MDA-MB-231 cells were purchased from the American Type Culture Collection (Manassas, VA, USA) and cultured in DMEM media containing 10% (v/v) fetal bovine serum and 100 units/ml of penicillin, 100 μg/ml of streptomycin. All cells were maintained at 37°C in a humidified incubator with an atmosphere of 5% CO_2_. For copper enrichment experiments, premalignant-MCF10 or MDA-MB-231 cells were cultured in media further supplemented with 25 μM CuCl_2 _for 3 days to 2 weeks. Whole cell extracts were prepared as described previously [29]. Briefly, cells were harvested, washed with PBS twice, and homogenized in a lysis buffer (50 mM Tris-HCI (pH 8.0), 150 mM NaCI, 0.5% NP40 (v/v), 0.5 mM phenylmethylsulfonyl fluoride, and 0.5 mM dithiothreitol) for 30 min at 4°C. Afterwards, the lysates were centrifuged at 12,000 g for 30 minutes, and the supernatants were collected as whole cell extracts.

### Color change and precipitate formation reactions

CQ, PDTC, TM, and CuCl_2 _were dissolved in DMSO to a final concentration of 50 mM. Then CuCl_2 _was mixed with each in a 1:1 ratio and qualitatively examined for color change and precipitate formation. After mixing, solutions were heated and vortexed repeatedly until clear. For the visual studies, solutions were examined for color change and precipitation as indicators of complex formation. In cellular studies, however, stock concentrations were kept lower (10 and 20 mM) prior to dilution during mixing in order to prevent precipitation.

### Cell proliferation assay

The MTT assay was used to determine the effects of these agents on overall proliferation of cells. Cells were plated in a 96-well plate and grown to 70–80% confluency, followed by addition of each compound at an indicated concentration for 24 h. MTT (1 mg/ml) in PBS was then added to wells and incubated at 37°C for 4 h to allow for complete cleavage of the tetrazolium salt by metabolically active cells. Next, MTT was removed and 100 μl of DMSO was added, followed by colorimetric analysis using a multilabel plate reader at 560 nm (Victor^3^; PerkinElmer (Wellesley, MA, USA)). Absorbance values plotted are the mean from triplicate experiments.

### Cellular and nuclear morphology analysis

A Zeiss (Thornwood, NY, USA) Axiovert 25 microscope was used for all microscopic imaging with either phase contrast for cellular morphology or fluorescence for nuclear morphology with Hoechst staining. For fluorescent nuclear morphology analysis, Hoechst stain was used as follows. Cells, either attached in plates or collected as a detached fraction, were washed once with ice cold PBS. Cells were then fixed in ethanol for 1 h and afterwards washed with ice cold PBS. Cells were stained with 50 μM Hoechst and kept in the dark at 4°C for 30 minutes and then visualized using fluorescence microscopy. Punctate and bright staining, or granular and bright staining nuclei were considered apoptotic.

### Copper pretreatment and ligand post-treatment

To simulate the *in vivo *copper status of cancer cells, premalignant-MCF10 and MDA-MB-231 cells were cultured in media containing 25 μM copper as done previously with prostate PC-3 cells [42]. MDA-MB-231 cells were cultured for a minimum of 48 h and premalignant-MCF10 cells were cultured for a minimum of 2 weeks. After copper enrichment culturing, cells were washed with PBS and then treated for the indicated hours using standard cell media containing TM (25 μM), CQ (1 to 100 μM), or PDTC (1 or 10 μM).

### Cellular copper measurement

Premalignant-MCF10 cells were cultured for 2 weeks in culture media with or without 25 μM CuCl_2_. Cells were collected and counted to determine total cells in the sample. Samples were spun down, washed with PBS, and provided to Quantum Labs (Wixom, MI, USA) for graphite furnace analysis to determine total copper in each sample.

### Western blot analysis

Cells were treated as indicated (see Figure legends). Afterwards, cells were harvested and lysed. Cell lysates (50 μg) were separated by SDS-PAGE and transferred to a nitrocellulose membrane, followed by visualization using the enhanced chemiluminescence kit (Amersham Biosciences, Piscataway, NJ, USA). Western blot analysis was performed using specific antibodies to ubiquitin and PARP as described previously [36]. Proteasome inhibition was measured as accumulation of ubiquitinated proteins and apoptosis by cleavage of PARP [36].

### Analysis of the proteasome chymotrypsin-like activity in whole cell extracts

Whole cell extracts (10 μg) of cells treated as indicated were incubated for 60 minutes at 37°C in 100 μl of assay buffer (50 mM Tris-HCL, pH 7.5) with 40 μM of fluorogenic substrate for the proteasomal chymotrypsin-like activity. After incubation, production of hydrolyzed 7-amino-4-methylcoumarin (AMC) groups was measured using a Victor 3 Multilabel Counter with an excitation filter of 380 nm and an emission filter of 460 nm (PerkinElmer, Boston, MA, USA). Changes in fluorescence were calculated against non-treated controls and plotted with statistical analysis using Microsoft Excel™ software.

## Results

### CQ and PDTC spontaneously react with copper to form a new complex

**Figure 2:**
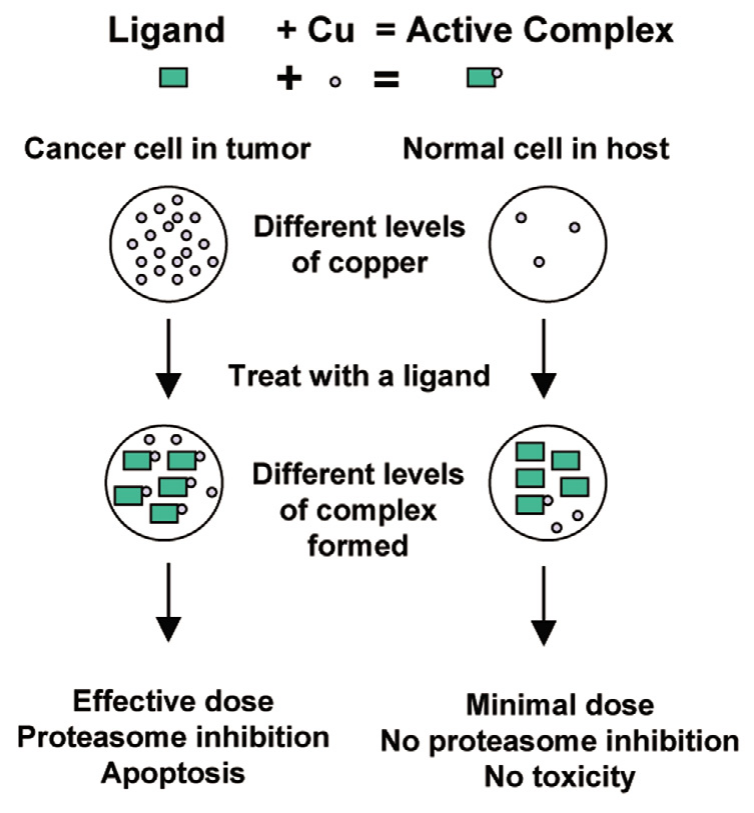
Diagram of a proposed copper-targeting therapeutic strategy. Cancer cells contain high levels of copper compared to normal cells. Upon treatment with a copper-binding ligand, a proteasome inhibiting copper complex will be formed. Only a minimal amount of complex should be formed in normal cells, therefore making them resistant to proteasome inhibition. In contrast, cancer cells may have a high dose of complex formed and are thus sensitive to proteasome inhibition, resulting in apoptosis. Copper forms the basis of the selection criteria between normal and tumor cells.

In order to use endogenous elevated tumor copper as a targeting mechanism for breast cancer therapy (Fig. 2), it is necessary that the ligand under consideration be capable of reacting spontaneously with copper to form a new complex. Complex formation reactions, particularly those involving metal, can result in dramatic color changes and/or precipitate formation. To test the reactivity of CQ and PDTC with copper, 50 mM of each was added to a 50 mM solution of copper (II) chloride (Fig. 1). The reaction of CQ and PDTC with copper, in DMSO, results in a dramatic color change (Fig. 1), indicating a chemical reaction has occurred and a metal complex has formed. These results are consistent with previous publications showing that both CQ and PDTC are strong copper chelators [51,55]. Therefore, these ligands may be capable of combining with endogenous tumor copper and forming a reactive complex.

The CQ-copper mixture has been further examined by the advanced photon source (APS) of Argonne National Laboratories (Argonne, IL, USA). The result is consistent with formation of a new complex between CQ and copper in solution (unpublished data). Furthermore, samples of a PDTC-copper mixture will be analyzed by the APS to confirm complex formation and the resulting structure. The details of these studies will be presented in a future manuscript.

### CQ and PDTC combine with copper to form proteasome-inhibitory complexes

**Figure 3:**
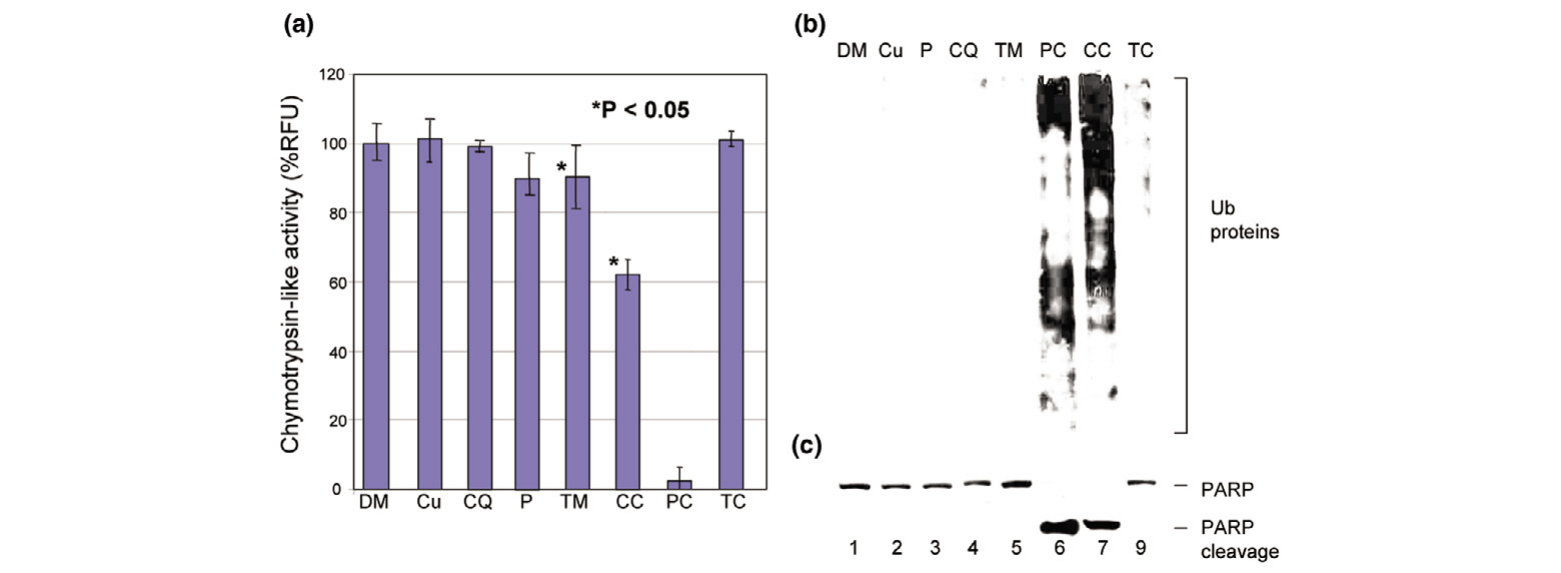
Inhibition of proteasome activity and apoptosis induction in MDA-MB-231 breast cancer cells by clioquinol (CQ) + copper and pyrrolidine dithiocarbamate (PDTC) + copper. MDA-MB-231 breast cancer cells were treated with 20 μM copper (Cu), CQ, CQ + copper (CC), tetrathiomolybdate (TM), TM + copper (TC), or 10 μM PDTC (P), or PDTC + copper (PC), using DMSO (DM) as a control. Cells were collected after 24 h treatment and analyzed for proteasome inhibition. **(a) **Proteasome activity as measured in released fluorescence units (RFUs) by release of 7-amino-4-methylcoumarins (AMCs) from substrate specific for chymotrypsin-like activity. **(b) **Western analysis for accumulation of ubiquitinated proteins as an indicator of proteasome inhibition. Treatment with PDTC + copper (PC; 10 μM) or CQ + copper (CC; 20 μM) results in reduced release of AMCs and ubiquitinated protein accumulation, suggesting proteasome inhibition. **(c) **Western analysis for cleavage of poly(ADP ribose) polymerase (PARP) as an indication of apoptosis. Treatment with CQ + copper (20 μM) or PDTC + copper (10 μM) results in cleavage of PARP, indicating that these complexes are capable of inducing apoptosis.

As both compounds can form a complex with copper, as indicated by color change (Fig. 1), we then tested whether these complexes were capable of inhibiting the proteasome activity in intact cells. Breast cancer MDA-MB-231 cells were treated with copper, CQ, CQ-copper mixture, PDTC, or PDTC-copper mixture, using TM and TM-copper mixture as controls. After a 24 h treatment, cells were collected and the cell extracts were prepared for analysis of proteasome inhibition by the chymotrypsin-like activity assay (Fig. 3a) and accumulation of ubiquitinated proteins (Fig. 3b). We found that both CQ-copper and PDTC-copper mixtures significantly inhibited the proteasome activity in MDA-MB-231 cells, as indicated by decreased levels of the proteasomal chymotrypsin-like activity (Fig. 3a) and accumulation of ubiquitinated proteins (Fig. 3b). The PDTC-copper mixture is more potent than that of CQ-copper (Fig. 3a). Copper, CQ, or PDTC alone had no effect. Interestingly, we found that TM and the TM-copper mixture had little to no proteasome-inhibitory activity (Fig. 3), supporting the inactive complex nature of TM-copper [42]. These data support that CQ and PDTC can combine spontaneously with copper to form a proteasome-inhibitory complex.

Although we have shown that copper alone can inhibit the activity of a purified proteasome [42], it is still possible that dithiocarbamates could be oxidized by copper to thiuram disulfides [59], which could be responsible for the observed proteasome inhibition. We therefore tested the effects of two thiuram disulfides and three disulfides on the proteasome activity. In the absence of copper, disulfiram (tetraethyl thiuram disulfide) and tetramethyl thiuram disulfide are incapable of inhibiting the proteasomal activity of MDA-MB-231 cell extract at micro-molar concentrations (data not shown). In addition, none of the tested disulfides, methyl propyl disulfide, allyl disulfide, and isopropyl disulfide, could inhibit the proteasome activity under the cell-free conditions (data not shown). This result suggests that complex formation between PDTC and copper, rather than general oxidation of PDTC to thiuram disulfide, is the likely mechanism of proteasome inhibition. Furthermore, we have found and reported that production of H_2_O_2 _does not occur in this system and that reductants do not block copper-mediated proteasome-inhibitory activity, supporting the idea that mechanisms other than oxidation are involved in proteasome inhibition [42]. This suggests that general oxidation or oxidation of dithiocarbamates is not sufficient to result in proteasome inhibition at these concentrations in these systems.

### CQ and PDTC when mixed with copper block proliferation of breast cancer MDA-MB-231 in a dose-dependent manner

**Figure 4:**
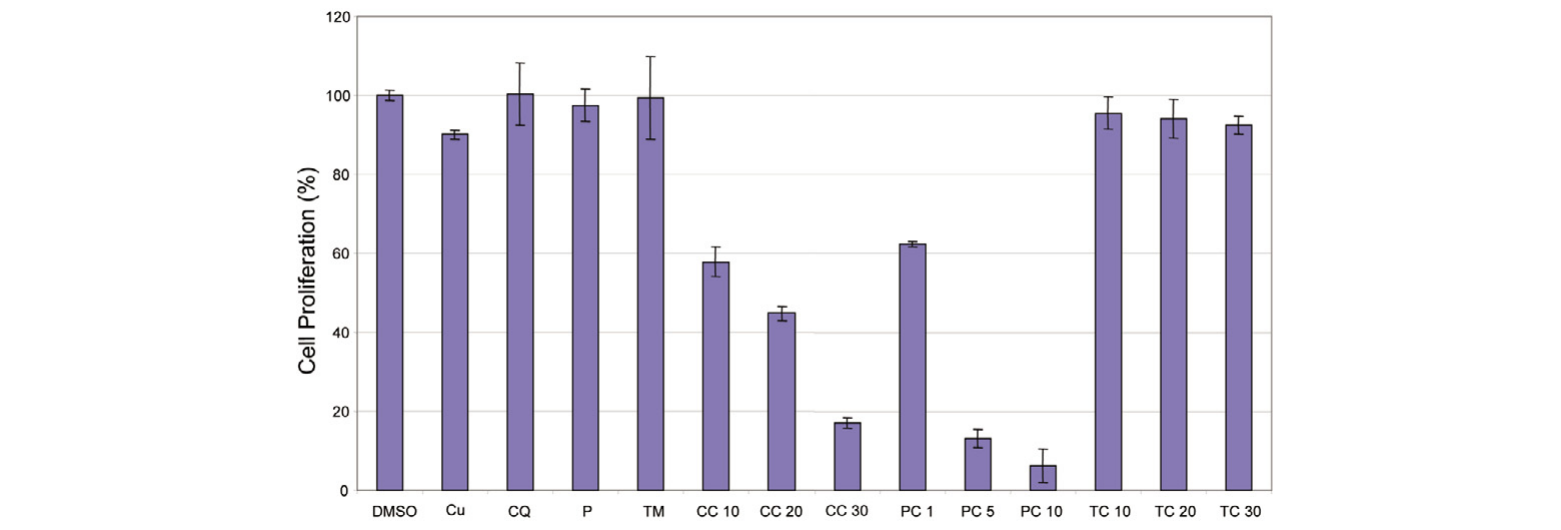
Anti-proliferative effects of clioquinol (CQ) + copper and pyrrolidine dithiocarbamate (PDTC) + copper. MDA-MB-231 cells were treated for 24 h with the following: copper (Cu; at 30 μM); CQ (CQ; at 30 μM); PDTC (P; 10 μM); tetrathiomolybdate (TM; 30 μM); CQ + copper (CC; 10, 20, 30 μM); PDTC + copper (PC; 1, 5, 10 μM); TM + copper (TC; 10, 20, 30 μM); or dimethylsulfoxide (DMSO) as a control. After 24 h the media was removed and cells were treated with 3-[4,5-dimethylthiazol-2-yl]-2,5-diphenyl-tetrazolium bromide (MTT) solution. Measurement of MTT conversion by absorbance at 560 nm showed that MDA-MB-231 cells responded in a dose-dependent manner to CQ-copper and PDTC-copper complexes. This suggests that these organic-copper complexes act as anti-proliferative agents.

After finding that CQ-copper and PDTC-copper mixtures could inhibit proteasome activity (Fig. 3a,b), we measured the effects of each compound on breast cancer cell proliferation (Fig. 4). We found that, associated with proteasome inhibition, the CQ-copper and PDTC-copper complexes inhibited cellular proliferation in a dose-dependent manner. CQ-copper showed 40% inhibition at 10 μM and increased to approximately 80% inhibition at 30 μM (Fig. 4). The PDTC-copper mixture inhibited proliferation by 40% at 1 μM and greater than 90% inhibition at 10 μM (Fig. 4). In contrast, copper, CQ, PDTC, or TM alone or TM mixed with copper had no significant effect (Fig. 4). The ranking of these compounds with respect to their ability to inhibit breast cancer cell proliferation matches well with their ability to inhibit the cellular proteasome activity (Figs. 4 versus 3a). Due to the nature of the MTT assay and the inability to separate apoptosis from growth arrest, both possible outcomes of the proteasome inhibition, IC_50 _values of these complexes were not measured. These data suggest that CQ and PDTC can spontaneously combine with copper to form an anti-proliferative complex.

### CQ and PDTC combine with copper to form a product toxic to malignant-MCF10 and MDA-MB-231 and premalignant-MCF10 cells, but non-toxic to normal-MCF10 breast cells

We found that the same CQ-copper and PDTC-copper complexes capable of proteasome inhibition (Fig. 3a, b) also demonstrated apoptosis induction, as shown by cleavage of PARP (Fig. 3c). In the absence of copper, neither CQ nor PDTC was able to induce apoptosis at these concentrations (Fig. 3c, lanes 6 and 7 versus lanes 3 and 4). TM, in the presence or absence of copper, does not induce apoptosis, further supporting TM's action as passive chelation and elimination of copper. These data support the idea that CQ and PDTC, but not TM, can combine spontaneously with copper to form a proteasome-inhibitory and apoptosis-inducing complex.

**Figure 5:**
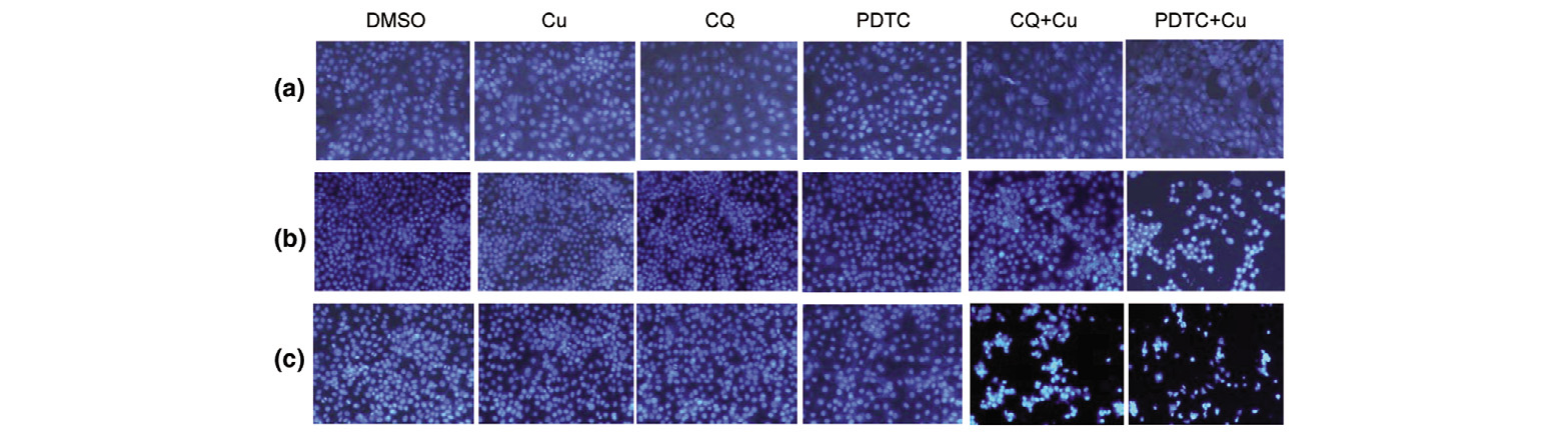
Induction of apoptosis by clioquinol (CQ) + copper and pyrrolidine dithiocarbamate (PDTC) + copper complexes in premalignant- and malignant-MCF10 cells. CQ and PDTC were mixed in a 1:1 molar ratio with CuCl_2_. Normal-, premalignant, and malignant-MCF10 cells were treated with CQ alone (CQ), copper alone (Cu), or CQ + copper (CQ+Cu) at 20 μM, or PDTC alone or PDTC + copper (PDTC+Cu) at 5 μM for 24 h. Dimethylsulfoxide (DMSO) was used as a control. After treatment, cells were stained with Hoescht for determination of apoptotic nuclei. Nuclei that were punctate or granular and bright were considered apoptotic. **(a) **The normal MCF10 cell line showed no apoptosis induction from the ligand, copper, or mixture (<2% in all cases). However, both **(b) **the premalignant MCF10AT1K.cl2 and **(c) **the malignant MCF10DCIS.com cells showed a dramatic induction of apoptotic nuclei after treatment with the mixture. **(b) **The percentages of apoptosic nuclei in MCF10AT1K.cl2 cells were: CQ+Cu, 10%; PDTC+Cu, 63%; and others <2%. **(c) **The percentages of apoptotic nuclei in MCF10DCIS.com cells were: CQ+Cu, 65%; PDTC+Cu, 75%; and others <2%.

To determine whether the CQ-copper and PDTC-copper complexes have differential effects on normal and tumor breast cells, the MCF10 series of cells [58] were then treated with CQ alone, copper alone, or the product of a 1:1 mixture of each at 20 μM (Fig. 5). The 20 μM CQ-copper complex induces apoptotic nuclei within 24 h for both premalignant- and malignant-MCF10 cells (10% and 65%, respectively; Fig. 5b, c). The malignant-MCF10 cells fully detached, suggesting that these cells were more sensitive to the complex than the premalignant cells. However, the normal-MCF10 cells demonstrated no apoptotic nuclei after 24 h of treatment with the CQ-copper complex (<2%; Fig. 5a).

We then tested the effects of the PDTC-copper mixture. The three breast cell lines were treated with PDTC alone, copper alone, or their mixture at 5 μM for 24 h. Again, both the premalignant- (63%; Fig. 5b) and the malignant-MCF10 (75%; Fig. 5c) cells showed a dramatic induction of apoptotic nuclei after treatment with the mixture, while the normal-MCF10 cells (<2%; Fig. 5a) showed no apoptosis induction from the mixture. As a control, neither CQ alone, PDTC alone, nor copper alone had effect on any of these cell lines (<2% in all the cases; Fig. 5). These data suggest that CQ and PDTC can spontaneously bind with copper and that the resulting complex is an apoptosis inducer to premalignant and cancerous, but not normal/non-transformed, breast cells, suggesting that such a complex if formed in a normal cell would not be toxic, but would be toxic in tumor cells.

### CQ and PDTC in complex with copper do not inhibit proteasome activity in normal breast MCF10A cells

**Figure 6:**
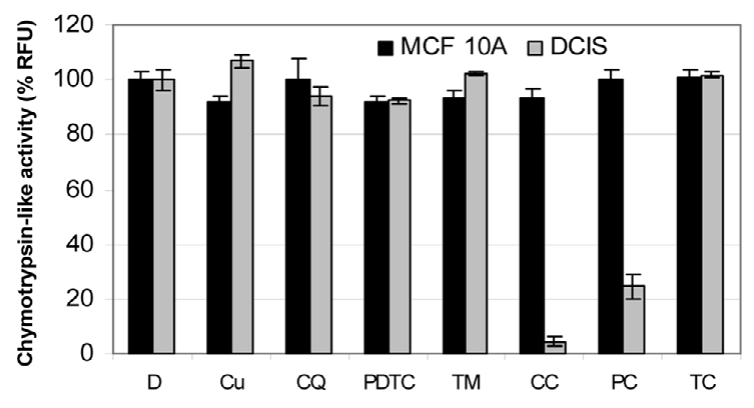
Clioquinol (CQ) + copper and pyrrolidine dithiocarbamate (PDTC) + copper fail to inhibit proteasome activity in normal MCF 10 (MCF-10A) cells. Normal MCF-10A and malignant mCF-10 (DCIS) breast cells were treated with 20 μM of copper (Cu), CQ, CQ + copper (CC), tetrathiomolybdate (TM), TM + copper (TC), or 5 μM PDTC or PDTC + copper (PC). Dimethylsulfoxide (D) was used as a control. After 24 h, cells were collected and lysed. Lysates were analyzed for chymotrypsin-like activity and showed as released fluorescence units (RFUs). The complexes were capable of eliminating proteasome activity in DCIS cells but not in MCF-10A cells. This strongly suggests that these metal complexes do not inhibit proteasome activity in normal breast cells.

To better understand the mechanism of resistance in normal breast cells to apoptosis induction by these organic-copper complexes, we treated both normal- and malignant-MCF10 cells with CQ-copper and PDTC-copper complexes and measured changes in the proteasome activity levels. Both cell lines were treated with 20 μM Cu, CQ, TM, CQ-copper, and TM-copper, or 5 μM PDTC and PDTC-copper (Fig. 6). We found that PDTC-copper and CQ-copper both strongly inhibited proteasome activity in malignant but not in normal cells (Fig. 6). Again, TM or the TM-copper mixture had no effects on either of the cell lines (Fig. 6). These data suggest that these organic-copper complexes do not inhibit the proteasome and, therefore, do not induce apoptosis in normal breast cells, further protecting normal cells from toxicity.

### Premalignant-MCF10 cells accumulate copper when cultured in copper-enriched conditions

A difficulty with examining the effectiveness of copper targeting in cell culture models is that cultured cancer cells seem to possess low to trace levels of copper [42]. This differs from the *in vivo *situation where cancer cells and tissues can contain micromolar concentrations of copper. In one study, the serum copper in breast cancer patients was approximately 2 μg/100 ml (equivalent to 0.3 μM) [60], while another study showed that the plasma copper levels in the malignant prostates were 124 μg/100 ml (equivalent to 19.5 μM) [16].

To simulate the *in vivo *situation, premalignant-MCF10 breast cells were cultured in media enriched with 25 μM CuCl_2 _for at least 2 weeks (see Materials and methods). Afterwards, cells were collected and subject to graphite furnace analysis to determine copper content (Table 1). The results of the analysis show that these cells can accumulate at least 16 times more copper when cultured in copper-enriched media (referred to here as copper-enriched cells) than when in a normal culture and an individual enriched cell has at least an order of magnitude more copper than a standard culture cell. Given a volume of 10 ml, this would be equivalent to 6 μM. Previously, we pretreated prostate cancer PC-3 cells with 100 μM CuCl_2 _for 48 h, which resulted in cellular copper levels being increased to 0.2 μM [42]. These data show that, in our enrichment system, premalignant-MCF10 cells can accumulate similar copper concentrations to those found in patients.

### Copper-enriched breast pre-malignant and cancer cells are sensitive to treatment with CQ or PDTC alone

Fundamental to the strategy we are presenting is the ability of a normally non-toxic ligand to bind with endogenous tumor cellular copper (Fig. 2). Studies in various cancer cells and tissues have found that patients can have copper concentrations in the micromolar ranges in those tissues [16,60]. Similarly, when premalignant-MCF10A cells are cultured in copper they can contain micromolar concentrations of copper (Table 1). We therefore tested the effects of CQ or PDTC alone in copper-enriched breast premalignant or cancer cells.

**Figure 7:**
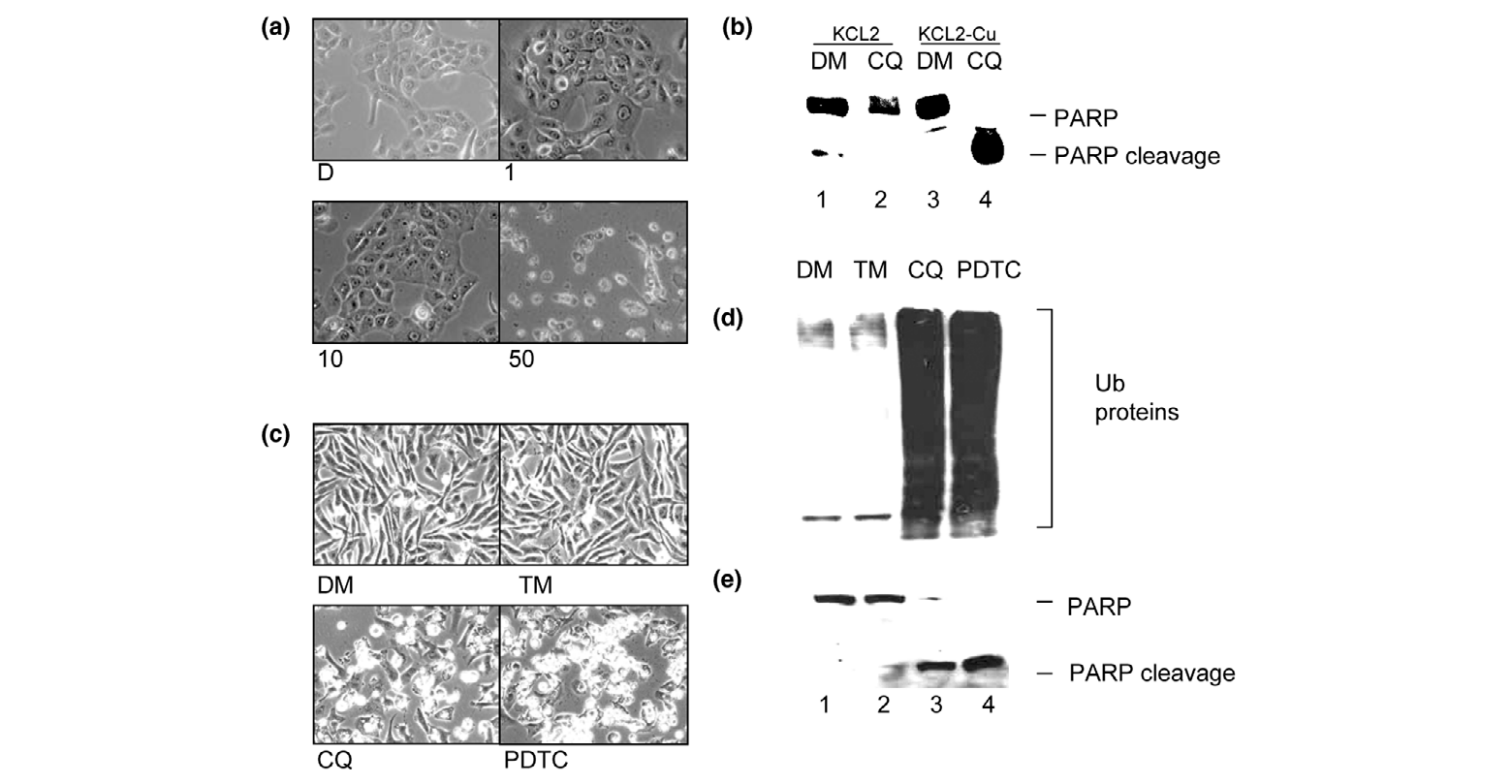
Induction of apoptosis in premalignant-MCF10 breast cells and proteasome inhibition and apoptosis induction in breast cancer MDA-MB-231 cells cultured to contain elevated copper and post-treated with clioquinol (CQ) and pyrrolidine dithiocarbamate (PDTC). **(a,c) **Premalignant-MCF10 (KCL2) cells were cultured in media containing 25 μM copper for two weeks. **(b,d,e) **MDA-MB-231 cells were cultured in 25 μM copper for 48 h. After culturing, cells were washed with PBS and treated with media containing: **(a) **1, 10, or 50 μM CQ; **(b-e) **25 μM CQ; 25 μM tetrathiomolybdate (TM); or **(b,d,e) **10 μM PDTC. An equivalent volume of DMSO (DM) was used as control. KCL2 and MDA-MB-231 cells were examined for **(a,b) **apoptotic morphology and **(c,e) **PARP cleavage. **(d) **MDA-MB-231 cells were also examined by western blot for accumulation of ubiquitinated proteins. **(a,c) **KCL2 cells containing clinically relevant levels of copper were sensitive to treatment by CQ alone, which induced apoptosis. **(c) **KCL2 cells cultured under standard conditions were resistant to treatment by 25 μM CQ. Similarly, MDA-MB-231 cells cultured to contain elevated copper were sensitive to CQ or PDTC and underwent proteasome inhibition as measured by accumulation of **(d) **ubiquitinated (Ub) proteins and apoptosis as evidenced by morphology and **(b,e) **poly (ADP ribose) polymerase (PARP) cleavage. These data suggest that KCL2 and MDA-MB-231 cells cultured to contain clinically relevant levels of copper are sensitive to treatment with CQ or PDTC alone, but not TM.

We first treated the copper-enriched premalignant-MCF10 cells with CQ alone. CQ at 1 to 10 μM caused apoptotic morphological changes of these copper-containing cells (Fig. 7a). Consistent with the morphology study, after 24 h treatment with CQ, these copper-enriched cells underwent extensive apoptosis, measured by the appearance of the PARP cleavage fragment (Fig. 7c). In contrast, premalignant-MCF10 cells that did not contain elevated copper were highly resistant to 25 μM of CQ (Fig. 7c, lanes 1 and 2). Similarly, copper-enriched premalignant-MCF10 cells were also sensitive to treatment with PDTC, but not TM (data not shown; Fig. 7b, d, e).

We also found that copper-enriched breast cancer MDA-MB-231 cells adopt apoptotic morphology after post-treatment with CQ or PDTC, but not TM (Fig. 7b). In the same experiment, lysates of these cells were subjected to western analysis. Both CQ and PDTC were capable of inducing proteasome inhibition and apoptosis in copper-pretreated MDA-MB-231 cells, as measured by accumulation of ubiquitinated proteins and cleavage of PARP, respectively (Fig. 7d, e). This is dramatically different from the behavior of these compounds in the absence of copper or in non-copper enriched cells (Fig. 7d, e versus Fig. 3b, c). In contrast, TM neither inhibited the proteasome activity nor induced apoptosis in these copper-enriched cells (Fig. 7b, d, e). These data support the idea that CQ and PDTC can spontaneously bind with copper in copper-enriched breast cancer cells and form an apoptosis-inducing complex and that cells containing trace or undetectable amounts of copper are resistant to this effect. It is possible, therefore, that CQ and PDTC act as apoptosis inducers through proteasome inhibition in a copper-dependent manner and can do so in cancer cells that contain copper in concentrations similar to those found in patients' tissues and serum.

## Discussion

A difficulty facing most cancer chemotherapy is the inability to discriminate between normal and malignant cells. Anti-angiogenesis and proteasome inhibition may be effective approaches to cancer therapy due to the dependence of cancer on these activities [9,37,61]. Unique among the trace metals, copper is required for angiogenesis [8-11]. Furthermore, it is well documented that cancer cells and tissues accumulate high concentrations of copper [13,18,21,62-64]. We previously reported that certain types of organic-copper complexes are capable of proteasome inhibition that is not a result of oxidative effects [42]. Therefore, the capability of organic copper to inhibit the proteasome, the necessity of copper for angiogenesis, and the accumulation of copper by cancer cells and tissues allows for a novel therapeutic strategy focusing on elevated copper as a selection mechanism against cancer cells and tissues (Fig. 2).

Our previous study [42] Additional file: 1 only briefly looked at an isolated system and examined the phenomenon of organic ligands binding to copper to form proteasome inhibitors and apoptosis inducers. The current study confirms and significantly expands our original findings. Specifically, this study examines a complete breast cancer system, including normal, premalignant and malignant cells. Furthermore, this study examines compounds that have clinical relevance and expands the copper enrichment studies. Several different approaches have been used in the analysis.

CQ and PDTC are two copper-binding compounds [51,53]. CQ has been investigated for use in Alzheimer's disease in regards to its ability to bind to copper found in amyloid plaques [43,47-50]. PDTC is a synthetic copper-binding antioxidant that has been studied for use in AIDS [53,54]. Previously, we have seen that 5,7-DiCl-8-OHQ (an analog of CQ) and PDTC when in complex with copper possessed strong proteasome-inhibitory and apoptosis-inducing abilities [42]. We report here the ability of CQ and PDTC to spontaneously react with copper, and inhibit the proteasome, which is followed by apoptosis, in breast cancer but not normal cells.

Our strategy revolves around the idea that a normally inactive or nontoxic organic ligand could bind with copper found in tumor tissues, resulting in a complex capable of proteasome inhibition. It has been shown that cancer cells are more sensitive to proteasome inhibition than normal cells [37,61,65-67]. To that end, we first verified that these two ligands directly interact with copper and form a new metal complex as indicated by dramatic color change (Fig. 1).

Once we verified that these two compounds spontaneously bind with copper and form a new complex we tested these complexes in MDA-MB-231 breast cancer cells to determine whether or not these complexes were proteasome inhibitors. We examined both cellular proteasome activity (Fig. 3a) and accumulation of ubiquitinated proteins (Fig. 3b). We found that treatment with ligand-copper mixtures significantly reduced chymotrypsin-like activity (Fig. 3a) and resulted in accumulation of ubiquitinated proteins (Fig. 3b), indicating proteasome inhibition had occurred. In contrast, ligand alone, copper alone, or TM mixed with copper did not inhibit the proteasome (Fig. 3). Previously we found that copper-mediated accumulation of ubiquitinated proteins is transient [42]. Therefore, the ubiquitinated protein pattern induced by CQ-copper and PDTC-copper shown in Fig. 3b should be considered transient and relevant only to the time point under consideration.

After determining that these organic-copper complexes could inhibit proliferation in MDA-MB-231 cells (Fig. 4), we examined the apoptosis-inducing abilities of the complexes. The organic-copper complexes were capable of inducing apoptosis strongly in malignant-MCF10 and MDA-MB-231, moderately in premalignant-MCF10, and did not induce apoptosis in normal-MCF10 cells (Figs. 5 and 3c). As a control, CQ, PDTC, TM or copper alone, or TM mixed with copper, were incapable of inducing apoptosis (Figs 5 and 3c). Therefore, the primary concerns of the strategy presented were fulfilled: the compound alone shows no toxic effects, the compound when mixed with copper becomes toxic, and the toxicity is limited to cancer cells and is associated with proteasome inhibition.

As these complexes have minimal to no effect on our normal cell line and seem to inhibit tumor cellular proteasome activity, we surmise that their toxicity to cancer cells stems from their proteasome inhibitory activity, to which normal cells are resistant. This was verified by examining proteasome activity in breast normal MCF10 cells compared to malignant-MCF10 cells (Fig. 6). We found that normal-MCF 10 cells did not suffer proteasome inhibition when treated with CQ or PDTC in complex with copper, although the concentrations tested inhibited the proteasome activity in malignant-MCF10 cells (Fig. 6), further supporting the argument that these complexes may be non-toxic to normal cells but are toxic to cancer cells through the mechanism of tumor-specific proteasome inhibition.

In a living organism, cancer cells and tissues accumulate high concentrations of copper [13,18,21,62-64]. To simulate this *in vivo *situation, premalignant-MCF10 and cancer MDA-MB-231 breast cells were cultured in copper-enriched media for either 2 weeks (premalignant-MCF10) or 72 h (MDA-MB-231). Afterwards, premalignant-MCF10 cells were collected and subjected to graphite furnace analysis to determine copper content. We found that premalignant-MCF10 cells were capable of accumulating concentrations of copper similar to those found in patient tissues (Table 1) and contained at least 16 fold more copper than cells cultured in standard media.

Once we had established cultures of premalignant-MCF10 cells enriched with copper, we then treated those cells with CQ or PDTC alone. Both CQ (25 μM) and PDTC (1 μM) induced apoptosis after treatment (Fig. 7a, c; data not shown). In cells cultured in enriched copper conditions, the compounds at similar concentrations had no effect (Figs 5 and 3c). Similarly, the breast cancer cell line MDA-MB-231, when cultured in elevated copper, is sensitized to apoptosis induction associated with proteasome inhibition with CQ and PDTC alone (Fig. 7b, d, e). This further supports our proposal that the compounds studied can use the increased copper load in cancer cells to form a proteasome inhibitor and an apoptosis inducer, whereas in the absence of this copper load these compounds have minimal to no effect at these concentrations.

The data presented here supports the novel concept of using accumulated copper in breast cancer cells and tissues as a selection method for chemotherapy. Non-toxic organic compounds such as CQ or PDTC can spontaneously bind with copper and form a proteasome inhibitor and an apoptosis inducer that has no effect on normal cells. Cancer cells, containing elevated copper, are sensitive to treatment with these organic compounds. Normal cells, containing trace amounts of copper, are resistant to these effects (Fig. 2). Both CQ and PDTC have been previously explored for use in other diseases and we believe these data support further investigation of these and other similar compounds in an anticopper/anticancer strategy. Most recently, another group also reported the anticancer activity of CQ [52]. Our data presented here may have provided a mechanistic interpretation for their findings.

The exact mechanisms of the copper-ligand combination are unclear at this time. However, it is apparent that cells cultured to contain elevated copper become sensitive to treatment with the ligands alone. We have future plans to work with the APS at Argonne National Laboratory to determine the final state of the ligand-copper complexes in cells. This should assist in further understanding why copper-enriched cells are sensitive to treatment with ligands that bind copper to form proteasome-inhibiting complexes. It should be noted that the system we have presented in this report is limited by looking at immortalized breast cancer cells rather than true normal primary cell lines. Future experiments should examine not only normal primary lymphocytes in culture but also animal studies to further confirm the effect on normal cells and tissues. Additional studies on cells that naturally contain elevated copper such as kidney, liver, and hematopoietic cells are also warranted.

## Conclusion

A unique feature of cancer cells is to accumulate high concentrations of copper [13,18,21,62-64]. We believe a potential strategy for cancer chemotherapy could involve the use of organic ligands that act as copper sensors and bind with the elevated copper in cancer cells and tissues. These complexes would act as proteasome inhibitors and apoptosis inducers to tumor cells. Because normal cells contain only trace amounts of copper, the organic ligands should form far fewer complexes with copper in them, thus exposing the normal cells to a minimal dose and reducing toxicity. We propose that treatment with copper-binding compounds such as CQ and PDTC will result in these compounds behaving as tumor 'sensors' using copper as a selection criterion. Therefore, this approach may convert the proangiogenic co-factor copper into a cancer-specific killing agent.

## Abbreviations

5,7-DiCl-8-OHQ = 5,7-dichloro-8-hydroxyquinoline; 8-OHQ = 8-hydroxyquinoline; APS = advanced photon source; CQ = clioquinol; DMEM = Dulbecco's modified Eagle medium; DMSO = dimethylsulfoxide; MTT = 3-[4,5-dimethylthiazol-2-yl]-2,5-diphenyl-tetrazolium bromide; PARP = poly(ADP ribose) polymerase; PBS = phosphate buffered saline; PDTC = pyrrolidine dithiocarbamate; TM = tetrathiomolybdate.

## Competing interests

The authors declare that they have no competing interests.

## Authors' contributions

KD and DC contributed equally to this manuscript. KD participated in the design of the study, data collection and interpretation, and manuscript preparation. DC participated in study design, data collection and interpretation, and manuscript preparation. SO and QCC participated in data collection. FRM participated in the design of the study, data interpretation, and manuscript preparation. QPD was responsible for the design of the study, data interpretation, and manuscript preparation as well as supervision of this project. All authors have read and approved the final manuscript.

## Supplementary Material

Additional File 1 A PDF file of reference [42]. Click here for file

## Figures and Tables

**Table 1:** Accumulation of copper by MCF10AT1K.cl2 cells

Cell line	No. of cells in sample	Total copper (μg)	Copper per cell
KCL2	5,725,000	ND	(1.75 × 10^-8^)^a^
KCL2-Cu25	1,468,750	0.4	2.72 × 10^-7^

^a^This is theoretical content assuming no more than 0.1 μg total copper, which is the minimal detection limit. Because cells grown in media without copper enrichment had no detectable copper, the number reported is the highest possible content per cell based on the minimum detection. ND, not detected.
