# Supplementary material for: Clioquinol and pyrrolidine dithiocarbamate complex with copper to form proteasome inhibitors and apoptosis inducers in human breast cancer cells
Source: Breast Cancer Res. 2005 Sep 20;7(6):R897–908. doi: 10.1186/bcr1322 (PMC1410741; doi:10.1186/bcr1322)
Supplement: Additional File 1 — A PDF file of reference [42]. [file bcr1322-S1.pdf]

Available online at www.sciencedirect.com
SCIENCE DIRECT®
Biochemical Pharmacology 67 (2004) 1139–1151
Biochemical Pharmacology
www.elsevier.com/locate/biochempharm

Organic copper complexes as a new class of proteasome inhibitors and apoptosis inducers in human cancer cells

Kenyon G. Daniel[a], Puja Gupta[a], R. Hope Harbach[b], Wayne C. Guida[a,b], Q. Ping Dou[a,*]

[a]Drug Discovery Program, Departments of Biochemistry & Molecular Biology and Interdisciplinary Oncology, H. Lee Moffitt Cancer Center & Research Institute, College of Medicine, University of South Florida, Tampa, FL, USA
[b]Department of Chemistry, Eckerd College, St. Petersburg, FL, USA

Received 18 June 2003; accepted 31 October 2003

Abstract

Here we report that organic copper complexes can potently and selectively inhibit the chymotrypsin-like activity of the proteasome *in vitro* and *in vivo*. Several copper compounds, such as NCI-109268 and bis-8-hydroxyquinoline copper(II) [Cu(8-OHQ)$_2$], can inhibit the chymotrypsin-like activity of purified 20S proteasome. In human leukemia cells, proteasome inhibition occurs within 15 min after treatment, followed by apoptosis. Neither proteasome inhibition nor apoptosis occurs in non-transformed, immortalized human natural killer cells under the same treatment. Furthermore, proteasome inhibition and apoptosis induction were detected in prostate cancer cells treated with the ligand 8-OHQ alone following pre-treatment with copper(II) chloride. None of these events occurred in cells treated with copper(II) chloride alone, 8-OHQ alone (without growth in copper-enriched media), or nickel(II) chloride pre-treatment followed by 8-OHQ. Furthermore, we found that copper-mediated inhibition of purified 20S proteasome cannot be blocked by a reducing agent and that organic copper compounds do not generate hydrogen peroxide in the cells, suggesting that proteasome inhibition and apoptosis induction are not due to copper-mediated oxidative damage of proteins. Our results suggest that certain types of organic ligands could bind to tumor cellular copper, forming potent proteasome inhibitors and apoptosis inducers at copper concentrations found in tumor tissues.
© 2003 Elsevier Inc. All rights reserved.

Keywords: Copper; Anti-copper drugs; Chelator; Proteasome inhibitors; Drug discovery

1. Introduction

Apoptosis is a highly conserved cellular suicide program in multicellular organisms from worms to humans [1–4]. This cellular death program serves as a means to maintain multicellular organisms by discarding damaged and undesirable cells [2,5]. Faulty execution of apoptosis, including either excessive cell death or insufficient cell death, is a factor in many disease states including AIDS and cancer [2]. Apoptosis features several distinct events and morphological changes, such as loss of the mitochondrial membrane potential, proteolytic dismantling of cellular components, DNA fragmentation, and cellular condensation into apoptotic bodies that are removed by phagocytes [1,6]. As a distinct series of cellular pathways, apoptosis potentially offers unique targets for chemotherapeutic intervention. It has been suggested that cancer cells are more sensitive to several apoptosis-inducing stimuli than

* Corresponding author. Present address: The Prevention Program, Department of Pathology, Barbara Ann Karmanos Cancer Institute, School of Medicine, Wayne State University, 516 HWCRC, 4100 John R Road, Detroit, MI 48201, USA. Tel.: +1-313-966-0641; fax: +1-313-966-7368.
E-mail address: doup@karmanos.org (Q.P. Dou).

Abbreviations: TM, tetrathiomolybdate; Trientine, triethylenetetramine; D-PA, D-penicillamine; Cu-TM, TM copper complex; Cu[trientine]Cl$_2$, trientine copper complex; Cu-D-PA, D-PA copper complex; 8-OHQ, 8-hydroxyquinoline hemisulfate; 8-OHQ-5-sulfonic acid, 8-hydroxyquinoline-5-sulfonic acid; 1,10-PT, 1,10-phenanthroline monohydrate; PDC, pyrrolidine dithiocarbamate; DBPTC, dibromo (1,10-phenanthroline)copper(II); DCPTC, dichloro (1,10-phenanthroline)copper(II); Cu[8-OHQ]$_2$, 8-OHQ copper complex, molar ratio 2:1; Cu[phen]Cl$_2$, 1,10-PT copper complex; 5,7-DCl, 5,7-dichloro-8-hydroxyquinoline; 5-*N*-8-OHQ, 5-amino-8-hydroxyquinoline; 8-OHQ-I-S, 8-hydroxy-7-iodo-5-quinoline-sulfonic acid; 8-OHI-Cu, 5-iodo-8-OHQ copper complex; 8-OHS-Cu, 8-OHQ-5-sulfate copper complex; (−)-EGCG, (−)-epigallocatechin-3-gallate; NAC, *N*-acetyl-L-cysteine; DMF, *N*,*N*-dimethylformamide; ICP-OES, inductively coupled plasma optical emission spectroscopy; PARP, poly(-ADP-ribose) polymerase; Ub, ubiquitin.

0006-2952/$ – see front matter © 2003 Elsevier Inc. All rights reserved.
doi:10.1016/j.bcp.2003.10.031

normal cells, including proteasome inhibitors and those affecting cellular division [7–9].

The proteasome is a massive multicatalytic protease responsible for degrading a large number of cellular proteins. These target proteins are first tagged with ubiquitin in order to be degraded by the proteasome. Several regulatory proteins involved in cell cycle and apoptosis processes, such as cyclins, bcl-2 family members, and p53, are degraded by the ubiquitin-proteasome pathway [10,11]. The eukaryotic proteasome possesses at least three distinct activities: chymotrypsin-like (cleavage after hydrophobic residues), trypsin-like (cleavage after basic residues), and caspase-like (cleavage after acidic residues) [12]. In a broad range of cell culture models, proteasome inhibitors rapidly induce tumor cell apoptosis, selectively activate the cell death program in oncogene-transformed, but not normal or untransformed cells, and are able to trigger apoptotic death in human cancer cells that are resistant to various anticancer agents [7–11]. Inhibition of the chymotrypsin-like, but not the trypsin-like, activity has been found to be associated with induction of tumor cell apoptosis [13,14]. The antitumor activity of proteasome inhibitors has been confirmed by the results of Phase I and II trials using MLN-341 (PS-341), a potent and selective dipeptidyl boronic acid proteasome inhibitor that recently gained FDA approval [15–17]. Phase III clinical trials for myeloma were conducted in spring 2003 [18]. However, some associated side effects were observed in the PS-341 trials [15,16], suggesting that there is a need to discover novel proteasome inhibitors with no, or much less, toxicity.

Tumor growth and metastasis depend upon angiogenesis, the neovascularization process [19,20] that requires growth factors, proteases, and the trace element copper [21–23]. Copper, but not other transition metals, is a cofactor essential for the tumor angiogenesis processes [21–23]. Consistently, high levels of copper have been found in many types of human cancers [24–27]. Copper stimulates proliferation and migration of human endothelial cells [28,29]. A specific amount of local copper appears to be required for angiogenesis to occur. It has been shown that three anti-copper drugs used in the treatment of Wilson's disease, TM, trientine, and D-PA, have anti-angiogenic effects in murine cancer models [30–32]. Based on this information, several clinical trials have been performed to evaluate the anti-angiogenic effects of these anti-copper drugs on solid tumors [21]. The anti-angiogenic effects of proteasome inhibitors have also been reported [33,34]. For example, treatment with the specific inhibitor lactacystin was previously shown to result in regions of avasculature in developing chick embryo chorioallantoic membrane, a common *in vivo* angiogenic assay [34]. However, the relationship between copper and the proteasome remains unclear.

Here we report that certain organic copper compounds are potent inhibitors of the proteasomal chymotrypsin-like activity. Furthermore, we have found that these organic compounds, different from traditional metal chelators, like EDTA, and anti-copper drugs, are capable of spontaneously forming active complexes with local copper in tumor cells. These organic copper complexes can inhibit proteasome activity and induce apoptosis. Further analysis revealed that the cause of these effects is likely not oxidative damage to proteins or DNA. These results suggest that use of copper-binding compounds to bind to local tumor or endogenous cellular copper, thus forming a copper complex, could function as a proteasome inhibitor strategy for chemotherapy.

2. Materials and methods

2.1. Materials

NCI-109268 was from the National Cancer Institute's Diversity Set Library. Fetal calf serum was purchased from Tissue Culture Biologicals. RPMI 1640 and Dulbecco's Modified Eagle Media (DMEM) were purchased from Invitrogen. Purified 20S rabbit proteasome was from BostonBiochem. A substrate for the chymotrypsin-like activity of the proteasome, Suc-Leu-Leu-Val-Tyr-AMC, was purchased from Calbiochem. A substrate for the trypsin-like activity of the proteasome, Z-Gly-Gly-Arg-AMC, was purchased from Bachem Bioscience Inc. Polyclonal anti-poly(ADP-ribose) polymerase (PARP) antibody was obtained from Boehringer, Mannheim. Monoclonal anti-ubiquitin antibody, secondary antibodies, anti-mouse IgG-horseradish peroxidase, and anti-rabbit IgG-horseradish peroxidase were from Santa Cruz Biotechnology Inc. Ethylenediaminetetraacetic acid (EDTA) was from Bio-Rad. *N*-Acetyl-leu-leu-norleucinal (LLnL), MG132, cupric bromide ($CuBr_2$), cupric chloride ($CuCl_2$), nickel chloride, manganese chloride, cobalt chloride, chromium chloride, ferrous chloride, cadmium chloride, zinc chloride, silver chloride, ammonium molybdate, magnesium sulfate, calcium chloride, cupric sulfate, cupric nitrate, NAC, dimethyl sulfoxide (DMSO), DMF, PDC, 8-OHQ, 8-OHQ-5-sulfonic acid, 5,7-DCl, 5-amino-8-hydroxyquinoline dihydrochloride (5-amino-8-OHQ), 5-iodo-8-hydroxyquinoline (5-iodo-8-OHQ), 8-hydroxy-7-iodo-5-quinoline-sulfonic acid (7-iodo-8-OHQ-5-sulfonic acid), trientine, D-PA, 1,10-PT, TM, and DBPTC, DCPTC, and (−)-EGCG were all purchased from Sigma-Aldrich.

2.2. Mixtures of organic copper compounds

One hundred millimolar solutions of $CuCl_2$ or $CuBr_2$ and 8-OHQ were made in DMSO or DMF as noted. Equal volumes of the 100 mM component solutions were mixed together to give a final soluble product at 50 mM. The soluble product was added to cells and an equal volume of solvent (DMSO or DMF) was used as a control. For experiments involving the pre-treatment of cells with

copper, both metal chloride and 8-OHQ were dissolved in water.

2.3. Synthetic chemistry

Synthetic Cu[8-OHQ]$_2$ [bis-8-hydroxyquinoline copper(II)] and copper–anti-copper complexes were dissolved in DMSO and heated at 95° for up to 5 min and vortexed vigorously until dissolved.

2.3.1. Cu[8-OHQ]$_2$

Use of 8-hydroxyquinoline to precipitate metal ions is well known [35]. We have utilized the procedure reported by Ohkaku and Nakamoto for the preparation of metal 8-hydroxyquinoline compounds [36]. Our procedure is effectively identical to the one reported by Fanning and Jonassen for the preparation of Cu[8-OHQ]$_2$ [37]. A solution of Cu(II)Cl$_2$·2H$_2$O (0.507 g, 2.97 mmol) in 15 mL of distilled water was added drop-wise to a solution of 8-hydroxyquinoline hemisulfate (1.228 g; 6.32 mmol) in 200 mL of 95% ethanol. The pH of the resulting mixture, which contained a greenish-brown precipitate, was adjusted to 7.0 by addition of 2 M aqueous ammonia and then the mixture was filtered and dried in a vacuum desiccator to yield 1.26 g of the complex (100% yield of crude material). The complex is only sparingly soluble in water. The IR spectrum was consistent with the one reported for Cu[8-OHQ]$_2$ [37]. The characterization by X-ray crystallography of Cu[8-OHQ]$_2$ prepared by precipitation of Cu(II) salts with 8-OHQ has previously been reported [38]. In the same manner, Cu(II) complexes of 8-OHI-Cu and 8-OHS-Cu were also prepared.

2.3.2. Cu[phen]Cl$_2$

The literature procedure [39] for the preparation of this complex was followed. A solution of Cu(II)Cl$_2$·2H$_2$O (0.449 g, 2.63 mmol) in 30 mL of 95% ethanol was added drop-wise to a solution of 1,10-PT (0.521 g, 2.63 mmol) in 50 mL of 95% ethanol. The resulting solution was allowed to cool to −20° in the freezing compartment of a refrigerator and the resulting precipitate was filtered. Concentration of the mother liquor produced a second crop of material to produce a total yield of 0.747 g (77%) of complex.

2.3.3. Cu[trientine]Cl$_2$

The procedure described by Siddiqi *et al.* was followed [40]. A solution of Cu(II)Cl$_2$·2H$_2$O (1.77 g, 10.3 mmol) in 20 mL of absolute ethanol was added drop-wise to a solution of trientine (1.53 g, 10.5 mmol) in 100 mL of absolute ethanol. Evaporation of the ethanol produced 3.30 g (100%) of complex.

2.3.4. Cu–TM complex (NH$_4$CuMoS$_4$) using Cu(II)Cl$_2$·2H$_2$O

A procedure identical to the one described previously by Lakshmanan *et al.* [41] was employed, with the exception that ammonium tetrathiomolybdate instead of piperidinium tetrathiomolybdate was used. A solution of Cu(II)Cl$_2$·2H$_2$O (0.569 g, 3.34 mmol) in 25 mL of absolute ethanol was added drop-wise to a hot suspension of (NH$_4$)$_2$MoS$_4$ (0.874 g, 3.36 mmol) in 125 mL of absolute ethanol. The pH of the resulting mixture was adjusted to 7.0 by addition of 3 M aqueous ammonia. The resulting mixture was allowed to cool to −20° in the freezing compartment of a refrigerator and the resulting mixture was filtered to yield 0.539 g (53%) of the complex. The presence of ammonium ion in our complex was confirmed by IR spectroscopy (3300, 1416 cm^{-1}) [42]. The literature [41,43] suggests that the complex prepared in this way actually contains Cu(I), with reduction of Cu(II) to Cu(I) presumably occurring during preparation of the complex.

2.3.5. Cu–D-PA complex

A procedure identical to the one described previously [44] was employed. D-Penicillamine (0.101 g, 0.677 mmol) was dissolved in 15 mL of sodium acetate buffer solution (0.5 M, pH 6.2). This solution was added to a solution of CuCl$_2$·2H$_2$O (0.0877 g, 0.516 mmol) in 2 mL of water. An equal volume of absolute ethanol was added to the resulting purple solution. The solution was cooled to 4° and the precipitate that was formed was filtered and dried in a vacuum dessicator to yield 0.108 g of material. Note that it is likely that the complex prepared in this way contains both Cu(I) and Cu(II), with oxidation of (some of the) PA to the disulfide occurring during preparation of the complex [44]. The complex presumably has the formula Na$_5$[Cu(II)$_6$Cu(I)$_8$(D-Pen)$_{12}$Cl]·nH$_2$O [44].

2.4. Cell culture, drug treatment, and cell extract preparation

Human leukemia cancer cell lines Jurkat T and HL-60 were cultured in RPMI media. Human prostate cancer PC-3 cells were maintained in DMEM. All media was supplemented with 10% fetal calf serum, 100 units/mL of penicillin, and 100 μg/mL of streptomycin. A human immortalized but non-transformed natural killer cell line (YT cells) [45] was maintained in RPMI growth media supplemented as above and further supplemented with 1 mM sodium pyruvate and 0.1 mM MEM non-essential amino acids (Invitrogen). All cell lines were maintained at 37° in a humidified incubator with an atmosphere of 5% CO$_2$.

Cells were treated for indicated times with noted molar amounts of compounds, mixtures, or with an equivalent volume of solvent (DMSO, DMF, H$_2$O) control as listed in individual experiments. For experiments requiring pre-treatment with metal, cells were grown in standard media containing 100 μM CuCl$_2$ or NiCl$_2$ as indicated. After 48 hr growth, metal supplemented media was removed and cells were washed twice with normal growth media and then continued to grow in normal growth media treated with 10 μM 8-OHQ or equivalent volume of solvent control.

A whole cell extract was prepared as described previously [46]. Briefly, cells were harvested, washed with PBS twice, and homogenized in a lysis buffer (50 mM Tris–HCl, pH 8.0, 150 mM NaCl, 0.5% NP-40, 0.5 mM phenylmethylsulfonyl fluoride, and 0.5 mM dithiothreitol) for 30 min at 4°. Afterwards, the lysates were centrifuged at 12,000 *g* for 30 min, and the supernatants were collected as whole cell extracts.

2.5. In vitro proteasome activity assay

The chymotrypsin-like activity of the proteasome was measured as previously described [47]. Briefly, purified 20S rabbit proteasome (0.10 μg) or Jurkat T cell extract (10 μg) was incubated with 20 μM fluorogenic peptide substrates, Suc-Leu-Leu-Val-Tyr-AMC (for chymotrypsin-like activity) or Z-Gly-Gly-Arg-AMC (trypsin-like activity), for 30 min at 37° in 100 μL of assay buffer (50 mM Tris–HCl, pH 7.5), with an inhibitor at indicated concentrations or equivalent volume of solvent as control. After incubation, production of hydrolyzed 7-amido-4-methyl-coumarin (AMC) groups was measured using a multi-well plate Wallac™ Victor^2 1420 Multilabel Counter with an excitation filter of 380 nm and an emission filter of 460 nm (Wallac). Changes in fluorescence were calculated against non-treated controls and plotted with statistical analysis using Microsoft Excel™ software.

2.6. Western blot analysis

Cell lysates (50 μg) were subject to SDS–PAGE and then transferred to a nitrocellulose membrane, followed by visualization *via* the enhanced chemiluminescence (ECL) kit (Amersham Biosciences). The ECL Western blot analysis was performed using specific antibodies to ubiquitin and PARP as described previously [13]. Proteasome inhibition was measured as accumulation of ubiquitinated proteins and PARP cleavage served as a marker for apoptosis [13].

2.7. Inductively coupled plasma optical emission spectroscopy (ICP-OES)

Analysis of the copper content of cells was performed with a Perkin-Elmer™ Optima 4300™ DV ICP-OES instrument. Cells were suspended in phosphate-buffered saline and diluted to 1–7 with doubly deionized water before analysis. Optical emissions were analyzed at three wavelengths specific for copper: 317.393, 324.752, and 224.700 nm.

2.8. Hydrogen peroxide (H_2O_2) detection

Jurkat T cells were treated with 5–10 μM of a solution prepared from the precipitation by adding $CuCl_2$ to 8-OHQ, or 5–10 μM of a mixture of $CuCl_2$ and 8-OHQ, or 100 μM of (−)-EGCG for up to 24 hr, followed by assaying the presence of H_2O_2 using Amplex® Red Hydrogen Peroxide Kit (Molecular Probes) in accordance with the manufacturer's directions.

3. Results

3.1. Organic copper compounds inhibit the chymotrypsin-like activity of the proteasome in vitro

To screen for new proteasome inhibitors, we analyzed the 1990 compounds in the NCI Diversity Set Library. Of them, NCI-109268 (Fig. 1A) was found to inhibit the chymotrypsin-like activity of the proteasome, although its efficacy was less than that of a known proteasome inhibitor LLnL (Fig. 1A) under these conditions (Fig. 1B). NCI-109268 inhibited the proteasomal chymotrypsin-like activity in both Jurkat T cell extract (Fig. 1B) and purified 20S rabbit proteasome (Fig. 1C), with an IC_{50} of ~6 μM. These results suggested that copper may play an important role in proteasome inhibition.

Inhibition of the proteasomal chymotrypsin-like, but not trypsin-like, activity is associated with induction of apoptosis in cancer cells [13,14]. Furthermore, certain proteasome inhibitors [i.e. (−)-EGCG] can also inhibit the caspase-like activity as well as the chymotrypsin-like activity of the proteasome [47]. Due to this, we limited our examination to the proteasomal chymotrypsin-like and the trypsin-like activities since that distinction was deemed the most important.

Owing to limited quantities of NCI-109268, we used a similar copper complex, Cu[8-OHQ]$_2$ (see Figs. 1A, 3 and 4), and determined its potency to the proteasomal chymotrypsin-like activity. In the first experiment, 8-OHQ was mixed with $CuCl_2$ at 1:1 molar ratio to generate the soluble product of 8-OHQ and $CuCl_2$. We found that the mixture of 8-OHQ and $CuCl_2$ had much greater inhibitory effect on the proteasomal chymotrypsin-like activity than the trypsin-like activity (Fig. 1D). Surprisingly, the salt $CuCl_2$ alone was also able to inhibit the chymotrypsin-like, and the trypsin-like activity though much more weakly, of the purified 20S proteasome (Fig. 1D). However, 8-OHQ alone was incapable of inhibiting either activity (Fig. 1D). These data suggest that copper is the essential component possessing the proteasome-inhibitory activity.

3.2. Copper, but not other metal, salts are inhibitors of the chymotrypsin-like activity of the proteasome in vitro

Since the central moiety of NCI-109268 is $CuCl_2$ and a common component of the compounds examined is copper (Fig. 1), we decided to examine the role of copper in proteasome inhibition. A series of metal salts with the same common oxidation state or from the same period as copper was tested for their capacity to inhibit the proteasome

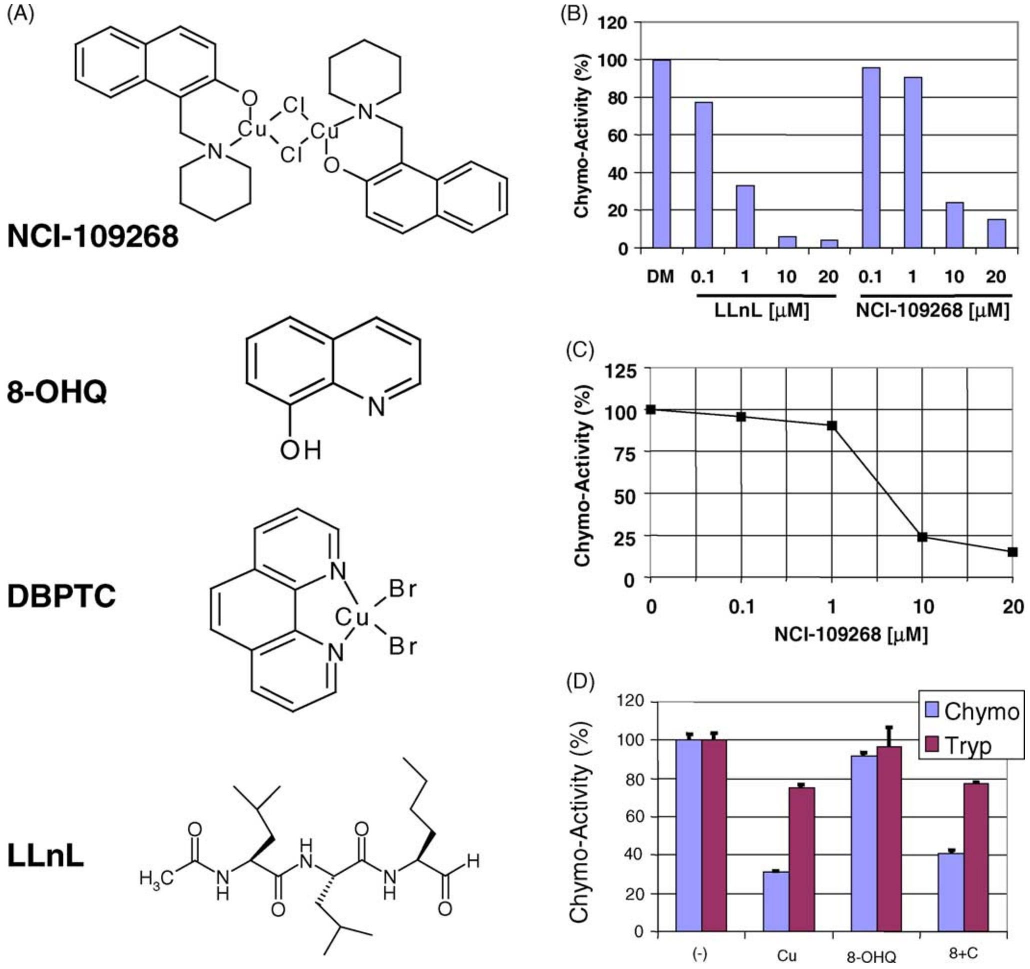

Fig. 1. Organic copper-mediated proteasome inhibition *in vitro*. (A) Structures of National Cancer Institute compound NCI-109268, 8-hydroxyquinoline (8-OHQ), dibromo-(1,10-phenanthroline)-copper(II) (DBPTC), and *N*-acetyl-L-leucinyl-L-leucinal-L-norleucinal (LLnL). (B) Concentration-dependent inhibition of proteasomal chymotrypsin-like activity in Jurkat cell extract (10 μg/reaction) by LLnL or NCI-109268 at the indicated concentrations as analyzed by release of AMC groups from substrate. An equivalent volume of DMSO (DM) is used as a negative control. (C) Analysis of concentration-dependent inhibition of purified rabbit 20S (0.10 μg) proteasomal chymotrypsin-like activity by NCI-109268. (D) Effects of $CuBr_2$ (Cu), 8-OHQ and soluble product of their mixture (8+C) (all at 10 μM) on chymotrypsin- and trypsin-like activities of purified 20S rabbit proteasome. (−) indicates no inhibitor was added.

activity using Jurkat T cell extract in an *in vitro* assay (Fig. 2A). Only $CuCl_2$ was found to inhibit the proteasomal chymotrypsin-like activity (Fig. 2A). To verify the role of copper, a series of copper salts was tested for inhibitory activity using Jurkat T cell extract. All of them were found to inhibit the proteasomal chymotrypsin-like activity with potency similar to that of the known proteasome inhibitor LLnL (Fig. 2B).

We next tested whether copper-mediated proteasome inhibition was a reversible or an irreversible (or tight binding) process. Purified 20S rabbit proteasome was incubated with $CuCl_2$ ranging from 1 to 10 μM. $CuCl_2$ by itself possessed an IC_{50} of ~3 μM (Fig. 2C). On the other hand, those reactions containing co-treatment of EDTA and copper resulted in no inhibition of proteasome activity, suggesting that EDTA, in this case, completely blocked copper's ability to inhibit the proteasome (Fig. 2C; "EDTA Co" compared to "No treatment"). However, if copper was permitted to react with the proteasome before the addition of EDTA, the IC_{50} of inhibition appeared to shift to approximately 8 μM (Fig. 2C; "EDTA Post"). After a longer exposure of the proteasome to $CuCl_2$, the addition of EDTA had no effect on the inhibitory nature of $CuCl_2$ and resulted in no shift of activity (data not shown). These results suggest that once the proteasome is inhibited by copper, the inhibition cannot be restored by metal chelation.

Previously it has been shown that under *in vitro* conditions, copper salts are capable of oxidizing some proteins (such as low density lipoprotein) and that this oxidation could be blocked by addition of NAC at 10–100 μM [48,49]. We reasoned that if indeed $CuCl_2$ causes oxidative damage to the purified 20S proteasome that could lead to inactivation of the proteasome, then NAC would be able to block the copper-mediated proteasome inhibition. To test this possibility, the *in vitro* proteasome activity assay was performed in the presence of 100 μM of NAC (Fig. 2D).

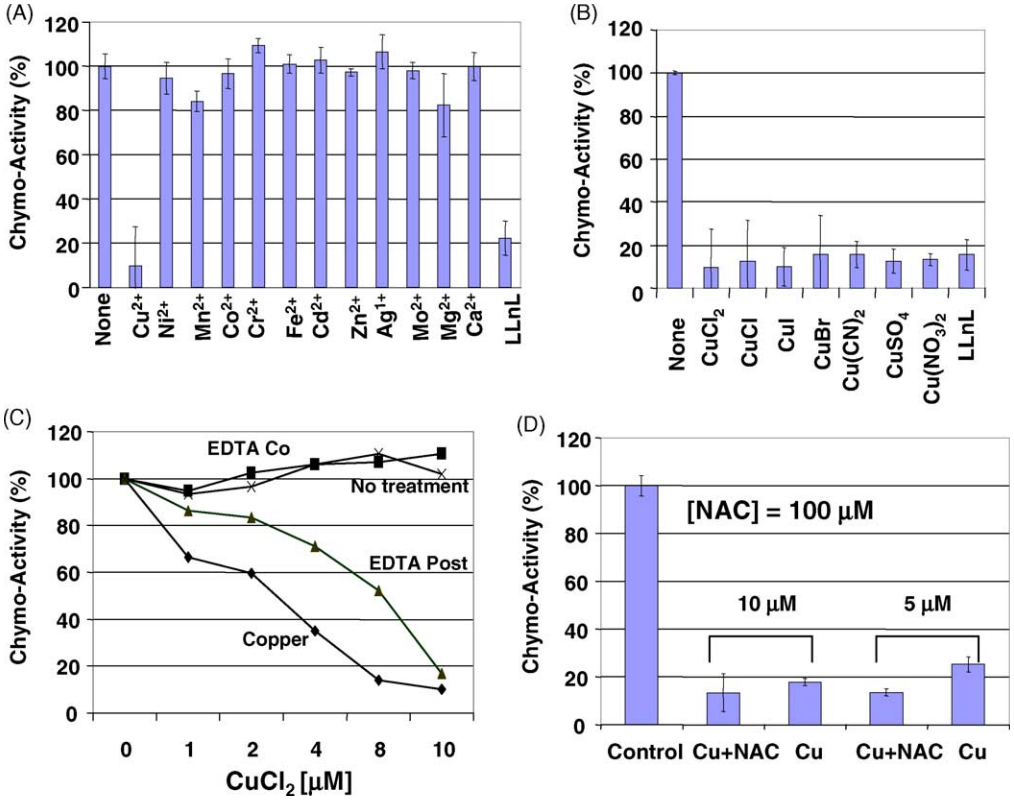

Fig. 2. *In vitro* inhibition of proteasomal chymotrypsin-like activity by inorganic copper. (A) Inhibition of chymotrypsin-like activity in Jurkat cell extract (10 μg/reaction) by 10 μM metal salts with the known proteasome inhibitor LLnL (10 μM) as control. (B) Effect of 10 μM copper (I and II) salts on chymotrypsin-like activity in Jurkat cell extract (10 μg/reaction). (C) The effects of EDTA (5 mM) on the chymotrypsin-like inhibitory activity of 10 μM $CuCl_2$ as a function of co-incubation (EDTA Co) or after 1 hr incubation of $CuCl_2$ (EDTA Post) with Jurkat cell extract (10 μg). (D) Effect of 100 μM NAC on inhibition of the chymotrypsin-like activity of purified rabbit 20S proteasome (0.10 μg) by 5 or 10 μM $CuCl_2$. "Cu + NAC" indicates co-incubation of NAC and $CuCl_2$; "Cu" indicates incubation with $CuCl_2$ but without NAC.

Co-incubation of $CuCl_2$ with NAC had no effect on the ability of copper to inhibit the purified 20S proteasome (Fig. 2D), suggesting that direct oxidative damage of the proteasome is not the cause of inhibition and it is likely that copper directly inhibits the proteasome.

3.3. Treatment with organic copper compounds, but not inorganic copper salts, results in proteasome inhibition and apoptosis in human leukemia cells

We then tested effects of inorganic and organic copper on the proteasome activity in intact tumor cells. Jurkat T cells were treated for 24 hr with 8-OHQ, $CuCl_2$, the mixture of 8-OHQ and $CuCl_2$, or the synthetic Cu[8-OHQ]$_2$ (see Section 2), all at 10 μM, followed by collecting the cells and measuring the proteasomal chymotrypsin-like activity in the prepared cell lysates (Fig. 3A). Treatment with 8-OHQ had no inhibitory effect on the proteasome activity levels measured using cell lysates, compared to the solvent-treated cells (Fig. 3A). Treatment with $CuCl_2$ inhibited ~30% of the proteasomal activity (Fig. 3A). In contrast, treatment with either the mixture or the synthetic Cu[8-OHQ]$_2$ completely inhibited the proteasome activity (Fig. 3A).

To investigate whether inhibition of the cellular proteasome activity is associated with induction of tumor cell death, trypan blue incorporation assay was performed in the same experiment (Fig. 3B). Treatment for 24 hr with the mixture or the synthetic Cu[8-OHQ]$_2$, but neither 8-OHQ nor $CuCl_2$ alone, induced loss of viability in Jurkat T cells (Fig. 3B).

To further investigate the *in vivo* effects of inorganic or organic copper, we mixed 8-OHQ with $CuBr_2$ or $Cu(NO_3)_2$ in DMF and obtained two different colored soluble products. When Jurkat T cells were treated with each of the two products, accumulation of ubiquitinated proteins, another measurement of proteasome inhibition [7–9], appeared as early as 15 min (Fig. 4A) and the apoptosis-specific PARP cleavage was apparent within 24 hr (Fig. 4B). The cells exposed to only copper salt or 8-OHQ alone did not undergo these changes (Fig. 4A and B). These data further suggest that some organic, but not inorganic, copper compounds can inhibit the proteasome and induce apoptosis in intact tumor cells.

Various other copper ligands, in addition to 8-OHQ, were tested as potential proteasome inhibitors when mixed with copper(II) salts. Jurkat T cells were treated with mixtures of $CuBr_2$ and 8-OHQ (8-OHQ $CuBr_2$), 5,7-dichloro-8-OHQ

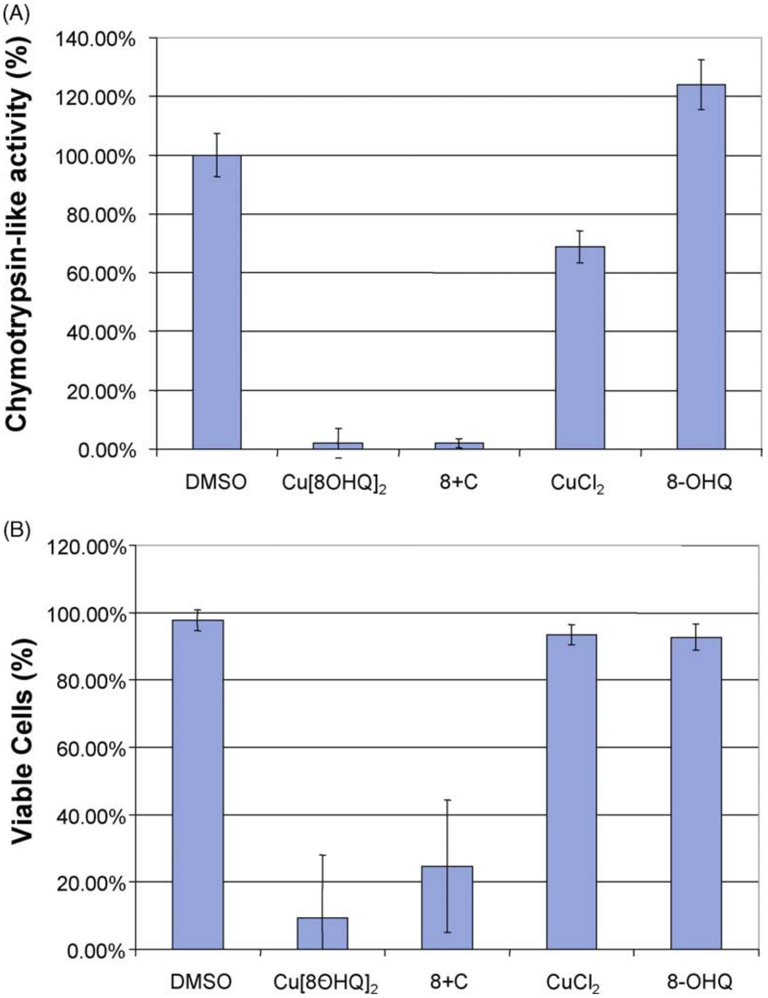

Fig. 3. Induction of cell death and inhibition of chymotrypsin-like activity in Jurkat T cells by synthetic copper complex and mixture. Jurkat T cells were treated with DMSO (DM), 10 μM of $CuCl_2$, 8-OHQ, the mixture (8+C; see Section 2), or synthetic Cu[8-OHQ]$_2$ for 24 hr and assayed for chymotrypsin-like activity (A) and cell viability (B).

(5,7-DCl 8-OHQ-Cu), pyrrolidinedithiocarbamate (PDC-Cu), or 5-amino-8-OHQ (5-*N*-8-OHQ-Cu), all at 10 μM, were able to accumulate ubiquitinated proteins at 15 min and induce PARP cleavage by 24 hr (Fig. 4C and D). The mixture of $CuBr_2$ and 7-iodo-8-hydroxy-quinoline-5-sulfate (8-OHQ-I-S-Cu) neither inhibited proteasome activity nor induced apoptosis (Fig. 4C and D).

Human leukemia HL60 cells were also treated with a commercial organic copper compound, DBPTC (Fig. 1A) for up to 10 hr. Levels of ubiquitinated proteins were increased by 15-fold at 1 hr, by 60-fold at 2 hr, and reached its maximal (150-fold) at 3 hr, which then remained high for up to 10 hr (80-fold; Fig. 4E). In the same experiment, PARP cleavage was detectable between 4 and 5 hr and increased at 10 hr (Fig. 4F), further supporting the conclusion that inhibition of the proteasome by organic copper occurs before apoptosis induction.

Next we synthesized several copper-containing complexes to compare their proteasome-inhibitory effects to the effects of the mixtures in Jurkat T cells. Similar to the results of the mixtures (Fig. 4A–D), both synthetic Cu[8-OHQ]$_2$ and DCPTC inhibited proteasome activity at 2 hr and caused apoptosis at 24 hr, while synthetic 8-OHI-Cu and 8-OHS-Cu were not able to do so at the same concentration (Fig. 4G and H).

Current anti-angiogenic therapies rely upon anti-copper compounds to complex copper making it unavailable for cellular use especially in angiogenesis [30–32]. In order to determine the possibility of an additional mode of action of anti-copper compounds involving proteasome inhibition, we tested whether the anti-copper drugs, TM, trientine and D-PA, when combined with copper, could also form proteasome inhibitors and apoptosis inducers. To do so, we synthesized complexes of Cu–TM, Cu–D-PA and Cu[trientine]Cl_2, and tested each of them in Jurkat T cells. None of these copper–anti-copper complexes induced either ubiquitinated protein accumulation or apoptosis, in contrast to the synthetic Cu[8-OHQ]$_2$ (Fig. 4G and H). These results reinforce the known activity of current anti-copper drugs as chelators of copper which block angiogenesis, and that

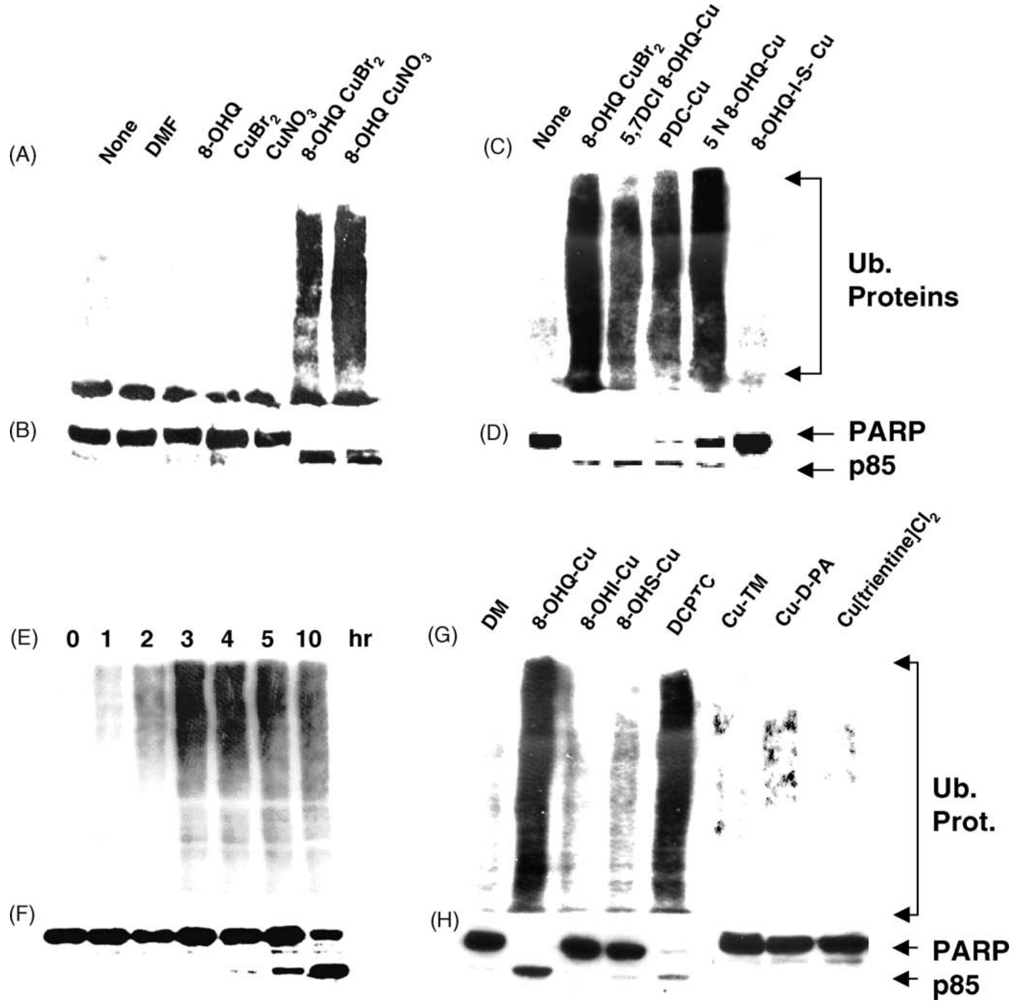

Fig. 4. Western Blot analysis of proteasome inhibition and apoptosis induction. Jurkat T cells (A–D, G, H) were treated with 10 μM of each indicated mixture, compound, or equivalent volume of solvent (DMF, DMSO) for control; for 15 min (A, C), 2 hr (G) or 24 hr (B, D, H). HL-60 cells (E, F) exposed to 50 μM of DBPTC for indicated hr. Western analysis was used to measure accumulation of ubiquitinated proteins (A, C, E, G) and PARP cleavage (B, D, F, H). (A, B) Mixtures of $CuBr_2$, $CuNO_3$, and 8-OHQ. (C, D) Mixtures of $CuBr_2$ and 8-OHQ, 5,7-dichloro-8-OHQ (5,7-DCl 8-OHQ-Cu), PDC (PDC Cu), 5-amino-8-OHQ (5-*N*-8-OHQ-Cu), or 8-OHQ-7-iodo-5-sulfonic acid (8-OHQ-I-S-Cu). (G, H) Synthetic Cu[8-OHQ]$_2$ (8-OHQ-Cu), 8-OHQ-7-iodo-copper(II) (8-OHI-Cu), 8-OHQ-5-sulfonic-acid-copper(II) (8-OHS-Cu), dichloro-1,10-phenanthroline-copper(II) (DCPTC), Cu–TM, Cu–D-PA, and Cu[trientine]Cl_2.

Cu[8-OHQ]$_2$ differs due to its ability to inhibit the proteasome and induce apoptosis in tumor cells.

It has been suggested that copper-mediated production of H_2O_2 is associated with cell death [50,51]. We then investigated whether organic copper complexes were also able to generate H_2O_2 prior to or during the process of apoptosis induction. Jurkat T cells were treated with either an aqueous solution of the precipitation prepared by adding $CuCl_2$ to 8-OHQ, a mixture of $CuCl_2$ and 8-OHQ, or a known peroxide producer (−)-EGCG [52,53], followed by measurement of levels of H_2O_2. All time points after copper treatment demonstrated no peroxide production (data not shown). As a positive control [52,53], treatment with 100 μM (−)-EGCG generated ∼8 μM H_2O_2 within 1 hr and remained high for up to 6 hr. This suggests that H_2O_2 production is probably not involved in these organic copper-mediated proteasome inhibition and apoptosis induction.

3.4. *Organic copper compounds exhibit selectivity in proteasome inhibition and apoptosis induction between transformed and non-transformed cell lines*

We then tested whether organic copper compounds could selectively induce proteasome inhibition and apoptosis in tumor or transformed, but not normal or nontransformed, cells. When leukemia Jurkat T cells were treated with a 1 μM 8-OHQ-Cu(II) mixture, ubiquitinated proteins accumulated at as early as 30 min, increased by 1 hr, and peaked at 3 hr (Fig. 5A), but decreased by 24 hr. In the same experiment, PARP cleavage was first detected at 3 hr and further increased at 24 hr (note the decreased levels of intact PARP protein and the disappearance of p85 fragment in Fig. 5B). In contrast, the same 8-OHQ-Cu(II) mixture treatment of non-transformed, immortalized natural killer cells (YT) resulted in only a slight accumulation of ubiquitinated proteins and did not induce apoptosis (Fig. 5A

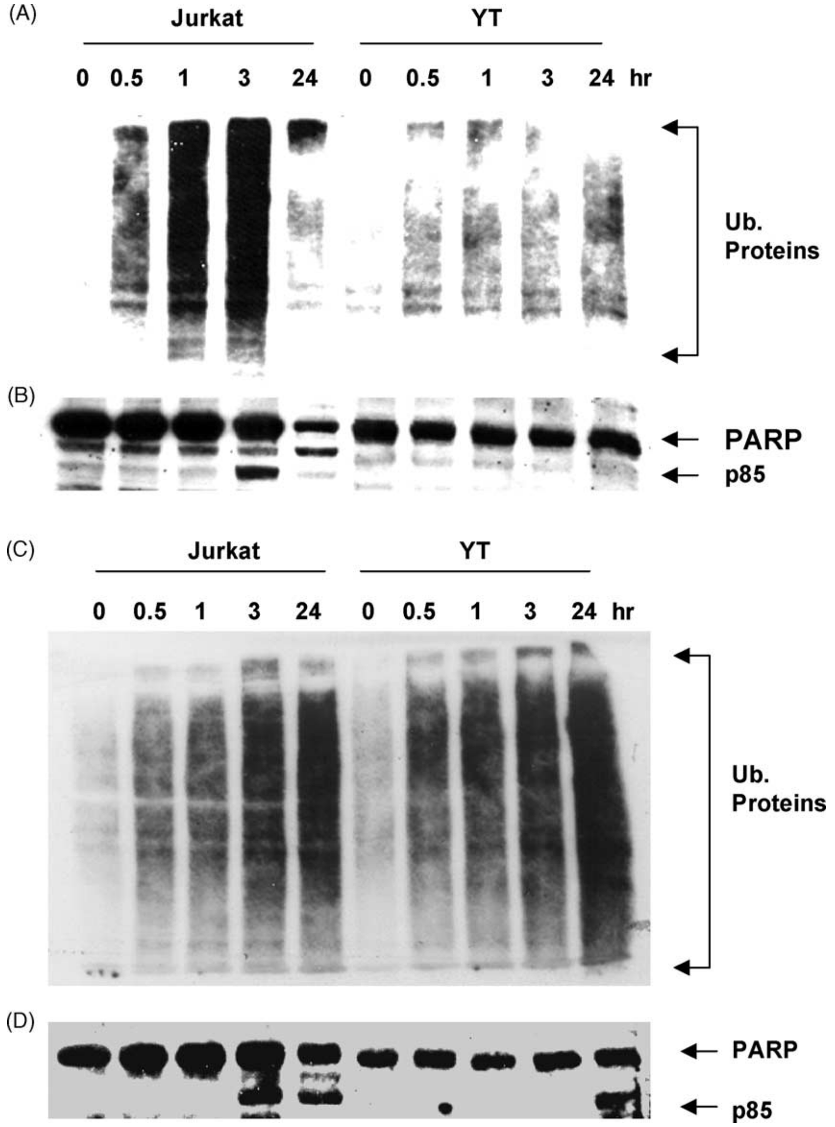

Fig. 5. Nontransformed cells are less sensitive to 8-OHQ $CuCl_2$ mixture than transformed cells. Leukemia Jurkat T and non-transformed NK (YT) cells were treated with 1 μM of 8-OHQ–copper(II) mixture (A, B) or the tripeptidyl proteasome inhibitor MG132 (C, D) for indicated hours, followed by Western analysis for levels of ubiquitinated proteins and appearance of PARP cleavage.

and B). In a similar experiment using the tripeptidyl proteasome inhibitor MG132, accumulation of ubiquitinated proteins and induction of apoptosis were found in both Jurkat T and YT cell lines, although YT cells were slightly more resistant to MG132 treatment than Jurkat T cells (Fig. 5C and D). These data suggest that organic copper compounds have selectivity between transformed and nontransformed cells, perhaps even more so than some traditional proteasome inhibitors.

3.5. *Tumor cells cultured in a copper-enriched environment are sensitive to treatment with a copper ligand*

Since tumor tissues typically possess higher levels of copper than normal tissues and anti-copper compounds are capable of binding local copper [30–32], we decided to test whether tumor endogenous cellular copper could interact with a ligand to form a proteasome inhibitor and an apoptosis inducer. Exponentially growing human prostate cancer PC-3 cells were cultured in media containing $CuCl_2$ [or $NiCl_2$ as control] for 48 hr and cellular copper concentrations increased from undetectable levels to ~165 nM, as determined by ICP-OES. $CuCl_2$ or $NiCl_2$ cultured PC-3 cells were then further incubated with standard media containing 8-OHQ. Of all the cells examined, only those pre-exposed to $CuCl_2$ followed by treatment with 8-OHQ demonstrated proteasome inhibition (from 15 min to 24 hr after 8-OHQ treatment), and apoptosis (PARP cleavage at 24 hr) (Fig. 6A and B). PC-3 cells treated with $CuCl_2$ or $NiCl_2$ alone, 8-OHQ alone, or $NiCl_2$ followed by 8-OHQ did not show ubiquitinated protein accumulation or PARP cleavage (Fig. 6A and B). These results support the hypothesis that 8-OHQ is capable of

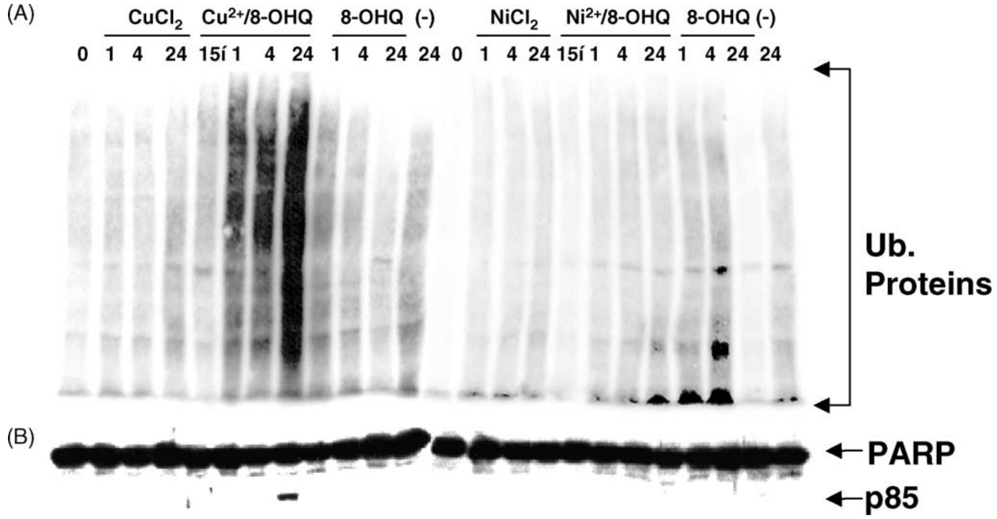

Fig. 6. Prostate cancer PC-3 cells cultured in copper-enriched media are sensitive to treatment with 8-OHQ. PC-3 cells were treated with 100 μM $CuCl_2$ or $NiCl_2$ for 48 hr achieving an intracellular copper concentration of ~165 nM, followed by 10 μM 8-OHQ (Cu^{2+}/8-OHQ) treatment for indicated hours. Continuous copper(II) or nickel(II) treatment ($CuCl_2$, $NiCl_2$), 8-OHQ treatment without copper(II) or nickel(II) pre-treatment, and no treatment [(−)] were used as controls. Samples were collected at the indicated time points and assayed by ICP-OES for cellular concentration of copper and Western analysis for accumulation of ubiquitinated proteins (A) and appearance of PARP cleavage (B).

binding endogenous tumor cellular copper, resulting in proteasome inhibition and apoptosis induction. Furthermore, these results suggest that the effective concentration of the 8-OHQ–copper complex must be less than 165 nM.

4. Discussion

Toxicity of many current chemotherapeutic drugs is partially due to their inability to distinguish normal cells from transformed cells. Of interest is the development of anti-angiogenic strategies and proteasome inhibitors. One proteasome inhibitor in clinical trials is PS-341. Phase I and II clinical trials confirmed the antitumor activity of PS-341 [15,16]. Studies showed that PS-341 was effective against a variety of tumor types either alone or in combinatorial treatment [18]. Phase III clinical trials for myeloma were conducted in spring 2003 [18]. The drug has gained FDA clearance and is in further clinical development for use against other forms of cancer [17]. However, the clinical trials also revealed its side effects [15,16], suggesting that further search for new proteasome inhibitors with no or little toxicity is necessary.

Towards the goal of discovering novel proteasome inhibitors as potential anticancer drugs, we screened the NCI Diversity Set of 1990 compounds with a fluorogenic *in vitro* activity assay. NCI-109268, a dimer of an organic copper complex, was found to inhibit the proteasomal chymotrypsin-like activity (Fig. 1).

Since the central moiety of NCI-109268 is copper chloride, we examined whether $CuCl_2$ itself and/or other transition metal salts were capable of inhibiting the chymotrypsin-like activity of the proteasome. The results showed that the copper ion, irrespective of its counter ion, was consistently capable of inhibiting the proteasome in a time-dependent and irreversible (or tight-binding) manner under *in vitro* conditions (Figs. 1 and 2). In addition, under *in vitro* conditions, the copper-mediated proteasome-inhibitory activity can be enhanced by certain ligands (such as 8-OHQ; data not shown) and blocked by strong copper chelators (such as EDTA; Fig. 2C). Furthermore, in intact cells, only certain organic copper compounds, but not inorganic copper salts, are able to strongly inhibit the proteasome activity, and the potency of organic copper complexes can be modified by altering its binding ligands (Figs. 3 and 4).

It has been reported that proteasome inhibitors are tumor cell apoptosis inducers [7–9,13,14]. We then examined whether or not organic copper complexes behaved the same way. We found that, much like other established proteasome inhibitors, organic copper compounds were only effective in inducing ubiquitinated protein accumulation and apoptosis in tumor, but not non-transformed cells (Fig. 5A and B). These results further suggested that the copper complexes are similar to other proteasome inhibitors and that the results are not due to general cellular toxicity.

Various human cancer cells contain increased levels of copper, an essential cofactor for tumor angiogenesis [24–27]. Without the copper-mediated tumor vascularization, tumors cannot grow or metastasize. It has been found that the cellular copper is not all protein-bound and can be stored in membranes [54]. A current strategy for cancer therapy is the inhibition of angiogenesis *via* copper control. Compounds developed for Wilson's disease, a copper storage disease, have recently been used to control the amount of copper found in tumor tissues as a method to prevent angiogenesis. We found that mixing an organic ligand such as 8-OHQ or 1,10-PT with a copper(II) salt

produced a soluble product that could inhibit the proteasome *in vitro* and *in vivo* and also induce apoptosis in intact tumor cells (Figs. 3 and 4 and data not shown). Next, we examined synthesized organic copper compounds based on our organic ligands and also based on current anti-copper drugs. This strategy allowed us to study comparatively these compounds to determine if current anti-copper drugs were also proteasome inhibitors and perhaps possessed an independent mode of action involving proteasome inhibition. We found that certain derivatives of quinoline in combination with $CuCl_2$ functioned similar to each other but quite different from complexes of known anti-copper drugs and copper (Fig. 4G and H). These results support the mechanism of the current anti-copper drugs as strong chelators of copper, rendering the copper ion unavailable for use by the cell. However, other organic ligands may be capable of binding copper in such a manner that it would possess proteasome inhibitory properties.

Furthermore, we tested whether or not the copper ligand 8-OHQ could interact with cellular copper and result in proteasome inhibition and apoptosis induction. Indeed, prostate cancer PC-3 cells grown in copper-enriched conditions (to generate cells bearing elevated copper as found in tumor tissues) are sensitive to treatment with ligand alone (Fig. 6). These data support the idea that copper in the tumor local cellular environment could be converted to a cell death inducer through the mechanism of proteasome inhibition by exposure to a copper-binding ligand (i.e. 8-OHQ). These data further suggest that the IC_{50} of such complexes would be in the nM range.

We hypothesize that the function of a copper ligand is to shield the copper from metabolic use by the cell, to aid delivery of the copper to the proteasome, and to increase the effectiveness of the copper-mediated proteasome-inhibitory activity, supported by the following arguments. First, the copper (but not other metal) salts tested were able to inhibit purified proteasome activity with potency similar to organic copper compounds (IC_{50} of 1–6 μM; Figs. 1 and 2 and data not shown). Second, both inorganic and organic copper compounds were capable of proteasome inhibition *in vitro* in a time-dependent and irreversible (or tight binding) manner (Figs. 1 and 2). Lastly, under *in vivo* conditions, only organic copper compounds, but not inorganic copper salts, were able to cause accumulation of ubiquitinated proteins and induction of apoptosis (Figs. 3–6).

Therefore, we hypothesize that the chemical nature of a copper ligand determines its capability of inhibiting the proteasome and inducing tumor cell apoptosis due to metabolic activities of the cell. Previous studies have shown that some organic copper compounds can function as DNA damaging agents [55,56]. However, under our experimental conditions we found that DNA strand breaks appeared several hours after proteasome inhibition and are a consequence of apoptosis (data not shown).

Since copper is a redox active element, there was concern that oxidation of proteins would result in upregulation of ubiquitination enzymes, resulting in the observed accumulation of ubiquitinated protein bands. We examined the possible production of H_2O_2 in Jurkat cultures after exposure to organic copper complexes. The accumulation of ubiquitinated proteins repeatedly occurred before or at 1 hr exposure, however, this was not accompanied by an increase in H_2O_2 (Figs. 4–6, and data not shown). Furthermore, the reducing agent NAC does not protect the proteasome from copper-mediated inhibition *in vitro* even when added simultaneously with copper (Fig. 2D).

Most recently, two groups have reported that a zinc(II)-dependent protease, POH1/Rpn11, a subunit of the 19S proteasome, is responsible for substrate deubiquitination during proteasomal degradation [57,58]. However, accumulation of ubiquitinated proteins by organic copper compounds does not appear to involve inhibition of deubiquitination of substrate proteins because a ligand alone has no effect (Figs. 3, 4 and 6) and a copper salt or an organic copper compound was able to inhibit the purified 20S proteasomal activity (Figs. 1 and 2). Indeed, the proteasomal enzymatic activity was inhibited in the cells treated with the mixture or synthetic copper compound (Fig. 3). Our data are consistent with the argument that copper is capable of directly inhibiting the proteasome. However, we realized that *o*-phenanthroline copper complexes are capable of oxidizing sulfhydryl groups on proteins [59] and ubiquitin hydrolases are sulfhydryl enzymes, whether quinoline–copper complexes can oxidize ubiquitin hydrolases requires further investigation.

A unique feature of cancer cells is to accumulate high concentrations of copper [24–27]. We believe that a strategy could be developed to bind this copper with specific ligands and form an anticancer agent within tumor tissues/cells. Cellular concentrations of copper in tumor cells and tissues should be high enough to promote complex formation. Tumors could be treated with copper ligands alone that would be non-toxic to cells, but could bind to tumor cellular copper, forming potent proteasome inhibitors. It is possible that the affinity of a copper ligand for copper-bearing cancer cells and the transient nature of the proteasome inhibition might protect normal cells from toxicity. As a result, we speculate that treatment with specific copper ligands, like the ones reported herein, will allow these normally innocuous compounds to become potent tumor cell-specific killers under copper-enriched conditions, which exist uniquely within many types of cancer cells and tissues. Therefore, this approach may convert copper, which is essential for tumor cell proliferation and angiogenesis, into a cancer cell-killing agent.

Acknowledgments

We thank NCI for providing the diversity set, Said Sebti, R. Chris Schnabel, David Smith, Aslamuzzaman Kazi, Audrey Burns, Sam Falsetti, and Michelle Blaskovich

for their help and discussion in this project; Michael Fedder for his assistance in preparing some of the copper compounds, and Ronald H. Goldfarb for providing YT cells. This work was supported in part by Moffitt Cancer Center & Research Institute.

References

[1] Jacobson MD, Weil M, Raff MC. Programmed cell death in animal development. Cell 1997;88:347–54.

[2] Song Z, Steller H. Death by design: mechanism and control of apoptosis. Trends Cell Biol 1999;9:M49–52.

[3] Earnshaw WC, Martins LM, Kaufmann SH. Mammalian caspases: structure, activation, substrates, and functions during apoptosis. Annu Rev Biochem 1999;68:383–424.

[4] Bratton SB, MacFarlane M, Cain K, Cohen GM. Protein complexes activate distinct caspase cascades in death receptor and stress-induced apoptosis. Exp Cell Res 2000;256:27–33.

[5] Wyllie AH, Kerr JF, Currie AR. Cell death: the significance of apoptosis. Int Rev Cytol 1980;68:251–306.

[6] Green DR, Reed JC. Mitochondria and apoptosis. Science 1998;281: 1309–12.

[7] Adams J. Potential for proteasome inhibition in the treatment of cancer. Drug Discov Today 2003;8:307–15.

[8] Dou QP, Li B. Proteasome inhibitors as potential novel anticancer agents. Drug Resist Update 1999;2:215–23.

[9] Almond JB, Cohen GM. The proteasome: a novel target for cancer chemotherapy. Leukemia 2002;16:433–43.

[10] Goldberg AL. Functions of the proteasome: the lysis at the end of the tunnel. Science 1995;268:522–3.

[11] Dou QP, Smith DM, Daniel KG, Kazi A. Interruption of tumor cell cycle progression through proteasome inhibition: implications for cancer therapy. In: Meijer L, Jezequel A, Roberge M, editors. Progress in cell cycle research. Life in Progress ed. Roscoff; 2003. p. 441–6.

[12] Lowe J, Stock D, Jap B, Zwickl P, Baumeister W, Huber R. Crystal structure of the 20S proteasome from the archaeon *T. acidophilum* at 3.4 A resolution. Science 1995;268:533–9.

[13] An B, Goldfarb RH, Siman R, Dou QP. Novel dipeptidyl proteasome inhibitors overcome Bcl-2 protective function and selectively accumulate the cyclin-dependent kinase inhibitor p27 and induce apoptosis in transformed, but not normal, human fibroblasts. Cell Death Differ 1998;5:1062–75.

[14] Lopes UG, Erhardt P, Yao R, Cooper GM. p53-dependent induction of apoptosis by proteasome inhibitors. J Biol Chem 1997;272:12893–6.

[15] Adams J. Preclinical and clinical evaluation of proteasome inhibitor PS-341 for the treatment of cancer. Curr Opin Chem Biol 2002;6: 493–500.

[16] Dou QP, Goldfarb RH. Bortezomib (Millennium Pharmaceuticals). IDrugs 2002;5:828–34.

[17] Twombly R. First proteasome inhibitor approved for multiple myeloma. J Natl Cancer Inst 2003;95:845.

[18] Adams J. The proteasome: structure, function, and role in the cell. Cancer Treat Rev 2003;29:3–9.

[19] Eatock MM, Schatzlein A, Kaye SB. Tumour vasculature as a target for anticancer therapy. Cancer Treat Rev 2000;26:191–204.

[20] Fox SB, Gasparini G, Harris AL. Angiogenesis: pathological, prognostic, and growth-factor pathways and their link to trial design and anticancer drugs. Lancet Oncol 2001;2:278–89.

[21] Brem S. Angiogenesis and cancer control: from concept to therapeutic trial. Cancer Control 1999;6:436–58.

[22] Brewer GJ. Copper control as an antiangiogenic anticancer therapy: lessons from treating Wilson's disease. Exp Biol Med (Maywood) 2001;226:665–73.

[23] Theophanides T, Anastassopoulou J. Copper and carcinogenesis. Crit Rev Oncol Hematol 2002;42:57–64.

[24] Scanni A, Licciardello L, Trovato M, Tomirotti M, Biraghi M. Serum copper and ceruloplasmin levels in patients with neoplasias localized in the stomach, large intestine or lung. Tumori 1977;63:175–80.

[25] Zowczak M, Iskra M, Torlinski L, Cofta S. Analysis of serum copper and zinc concentrations in cancer patients. Biol Trace Elem Res 2001; 82:1–8.

[26] Turecky L, Kalina P, Uhlikova E, Namerova S, Krizko J. Serum ceruloplasmin and copper levels in patients with primary brain tumors. Klin Wochenschr 1984;62:187–9.

[27] Chan A, Wong F, Arumanayagam M. Serum ultrafiltrable copper, total copper and caeruloplasmin concentrations in gynaecological carcinomas. Ann Clin Biochem 1993;30:545–9.

[28] McAuslan BR, Reilly W. Endothelial cell phagokinesis in response to specific metal ions. Exp Cell Res 1980;130:147–57.

[29] Hu GF. Copper stimulates proliferation of human endothelial cells under culture. J Cell Biochem 1998;69:326–35.

[30] Pan Q, Kleer CG, van Golen KL, Irani J, Bottema KM, Bias C, De Carvalho M, Mesri EA, Robins DM, Dick RD, Brewer GJ, Merajver SD. Copper deficiency induced by tetrathiomolybdate suppresses tumor growth and angiogenesis. Cancer Res 2002;62:4854–9.

[31] Yoshii J, Yoshiji H, Kuriyama S, Ikenaka Y, Noguchi R, Okuda H, Tsujinoue H, Nakatani T, Kishida H, Nakae D, Gomez DE, De Lorenzo MS, Tejera AM, Fukui H. The copper-chelating agent, trientine, suppresses tumor development and angiogenesis in the murine hepatocellular carcinoma cells. Int J Cancer 2001;94:768–73.

[32] Brem SS, Zagzag D, Tsanaclis AM, Gately S, Elkouby MP, Brien SE. Inhibition of angiogenesis and tumor growth in the brain. Suppression of endothelial cell turnover by penicillamine and the depletion of copper, an angiogenic cofactor. Am J Pathol 1990;137:1121–42.

[33] Sunwoo JB, Chen Z, Dong G, Yeh N, Crowl Bancroft C, Sausville E, Adams J, Elliott P, Van Waes C. Novel proteasome inhibitor PS-341 inhibits activation of nuclear factor-kappa B, cell survival, tumor growth, and angiogenesis in squamous cell carcinoma. Clin Cancer Res 2001;7:1419–28.

[34] Oikawa T, Sasaki T, Nakamura M, Shimamura M, Tanahashi N, Omura S, Tanaka K. The proteasome is involved in angiogenesis. Biochem Biophys Res Commun 1998;246:243–8.

[35] Kolthoff IMS, Sandell EB, Kolthoff IMS. Textbook of quantitative inorganic analysis. New York: The MacMillan Co.; 1959.

[36] Ohkaku N, Nakamoto K. Metal isotope effect on metal-ligand vibrations VI. Metal complexes of 8-hydroxyquinoline. Inorg Chem 1971;10:798–805.

[37] Fanning JC, Jonassen HB. The reaction of 8-quinolinol with copper(II) salts. J Inorg Nucl Chem 1963;25:29–35.

[38] Bevan J, Graddon D, McConnell J. Crystal structures of bis-(salicylaldehydato)-copper(II) and bis-(8-hydroxyquinolinato)-copper(II). Nature 1963;363.

[39] Ray N, Tyagi S, Hathaway B. The preparation and properties of [Cu(chelate)]X2 complexes. Crystal structure and electronic properties of bis(2-pyridylamine)dibromo-copper(II). J Chem Soc Dalt Trans: Inorg Chem 1982;143–6.

[40] Siddiqi KS, Aqra FMAM, Shah SA, Zaidi SAA. A transition metal complex as a ligand in the chemistry of group (IV) elements. Polyhedron 1993;12:949–53.

[41] Lakshmanan V, Nagaraja KS, Udupa MR. Synthesis and characterization of copper(I) tetrathiomolybdates. Ind J Chem 1994;33A: 772–4.

[42] Conley RT. Infrared spectroscopy. Boston: Allyn and Bacon; 1966.

[43] Binne WP, Redman MJ, Mallio WJ. On the preparation, properties, and structure of cuprous ammonium thiomolybdate. Inorg Chem 1970;9:1449–52.

[44] Paul JM, Birker WL, Freeman HC. Properties, and function of a copper(I)–copper(II) complex of D-penicillamine. J Am Chem Soc 1977;33:6890–9.

[45] Drexler HG, Matsuo AY, MacLeod RA. Continuous hematopoietic cell lines as model systems for leukemia–lymphoma research. Leuk Res 2000;24:881–911.

[46] An B, Dou QP. Cleavage of retinoblastoma protein during apoptosis: an interleukin 1 beta-converting enzyme-like protease as candidate. Cancer Res 1996;56:438–42.

[47] Nam S, Smith DM, Dou QP. Ester bond-containing tea polyphenols potently inhibit proteasome activity *in vitro* and *in vivo*. J Biol Chem 2001;276:13322–30.

[48] Godfrey EG, Stewart J, Dargie HJ, Reid JL, Dominiczak M, Hamilton CA, McMurray J. Effects of ACE inhibitors on oxidation of human low density lipoprotein. Br J Clin Pharmacol 1994;37:63–6.

[49] Rattan AK, Arad Y. Temporal and kinetic determinants of the inhibition of LDL oxidation by *N*-acetylcysteine (NAC). Atherosclerosis 1998;138:319–27.

[50] Chen SH, Liu SH, Liang YC, Lin JK, Lin-Shiau SY. Death signaling pathway induced by pyrrolidine dithiocarbamate–Cu(2+) complex in the cultured rat cortical astrocytes. Glia 2000;31:249–61.

[51] Sakano K, Oikawa S, Hasegawa K, Kawanishi S. Hydroxyurea induces site-specific DNA damage via formation of hydrogen peroxide and nitric oxide. Jpn J Cancer Res 2001;92:1166–74.

[52] Yang G, Liao J, Li C, Chung J, Yurkow E, Ho C, Yang C. Effect of black and green tea polyphenols on *c*-jun phosphorylation and H(2)O(2) production in transformed and non-transformed human bronchial cell lines: possible mechanisms of cell growth inhibition and apoptosis induction. Carcinogenesis 2000;21:2035–9.

[53] Sakagami H, Arakawa H, Maeda M, Satoh K, Kadofuku T, Fukuchi K, Gomi K. Production of hydrogen peroxide and methionine sulfoxide by epigallocatechin gallate and antioxidants. Anticancer Res 2001; 21:2633–41.

[54] Stockert RJ, Grushoff PS, Morell AG, Bentley GE, O'Brien HA, Scheinberg IH, Sternlieb I. Transport and intracellular distribution of copper in a human hepatoblastoma cell line, HepG2. Hepatology 1986; 6:60–4.

[55] Zelenko O, Gallagher J, Xu Y, Sigman DS. Chemical nuclease activity of 1,10-phenanthroline-copper. Isotopic probes of mechanism. Inorg Chem 1998;37:2198–204.

[56] Taenaka S, Ihara I, Takagi M. Cleavage of double helical DNA by Cu^{2+} ion in the presence of bisintercalator containing penta(ethylene glycol) connector chain. J Mol Recog 1990;3:156–62.

[57] Verma R, Aravind L, Oania R, McDonald WH, Yates III JR, Koonin EV, Deshaies RJ. Role of Rpn11 metalloprotease in deubiquitination and degradation by the 26S proteasome. Science 2002;298:611–5.

[58] Yao T, Cohen RE. A cryptic protease couples deubiquitination and degradation by the proteasome. Nature 2002;419:403–7.

[59] Kobashi K, Horecher B. Reversible inactivation of rabbit muscle aldolase by ophenanthroline. Arch Biochem Biophys 1967;121: 178–86.
